# The effect of adult psychological therapies on employment and earnings: Evidence from England

**DOI:** 10.1017/S003329172510305X

**Published:** 2026-02-27

**Authors:** Klaudia Rzepnicka, Emma Sharland, Marta Rossa, Ted Dolby, Ekaterina Oparina, Rob Saunders, Daniel Ayoubkhani, Vahé Nafilyan

**Affiliations:** 1 National Statisticians Analysis Unit, Office for National Statistics, UK; 2 Centre for Economic Performance, London School of Economics, UK; 3 CORE Data Lab, Centre for Outcomes and Research Effectiveness, Research Department of Clinical, Educational and Health Psychology, University College London, UK

**Keywords:** earnings, employment, labor market outcomes, mental health, NHS Talking Therapies, psychological therapies

## Abstract

**Background:**

People suffering from common mental disorders (CMDs), such as depression and anxiety, are more likely to be inactive in the labor market. Psychological therapies are highly effective at treating CMDs, but less is known about their impact on long-term labor market outcomes.

**Methods:**

Using national treatment program data in England, NHS Talking Therapies (NHSTT), with unique linkage to administration data on employment and census records, we estimated the effects of NHSTT on employment and earnings. We used an event study approach using individual fixed effects to capture time-invariant confounders and natural recovery.

**Results:**

Overall, completing treatment led to a maximum average increase of £17 in monthly earnings (year 2) and a likelihood of paid employment by 1.5 percentage points (year 7). Those ‘Not working, seeking work’ saw a maximum average increase in pay of £63 per month (year 7) and a likelihood of paid employment by 3.1 percentage points (year 4). Patients in the younger age groups (25–34 years) saw the largest effect on the likelihood of paid employment by 2.3 percentage points (year 7), followed by those aged 35–44 years with 2.0 percentage points (year 5).

**Conclusions:**

Completion of psychological treatment for CMDs through the national NHSTT program leads to sustained increases in both employment and earnings up to 7 years after the start of treatment. Our findings demonstrate the economic benefits of treating CMDs and how investing in mental health can impact labor market participation.

## Background

Globally, common mental disorders (CMDs) such as depression and anxiety are among the most commonly reported conditions among people who are neither working nor looking for work due to long-term sickness (Casimirri et al., 2014; Collins et al., 2005; Nexo et al., 2018; OECD, 2012; Office for National Statistics, 2023b). Individuals with CMDs are more likely to be unemployed, experience longer durations of unemployment and long-term sickness absences, have lower incomes, and be at higher risk of poverty (Butterworth, Leach, Pirkis, & Kelaher, 2012; Casimirri et al., 2014; Nexo et al., 2018; OECD, 2012). Previous reports have estimated that poor mental health costs the economy around £110 billion a year (Cardoso & McHayle, 2024), accounting for ~5% of UK Gross Domestic Product (GDP) in 2019 (Mcdaid et al., 2022). Increasing trends of economic inactivity and rising rates of CMDs point to a growing impact of mental ill-health on the economy (Office for National Statistics, 2022a).

In recent years, governments across the world have made efforts to mitigate the adverse effects of CMDs by increasing funding for mental health care and facilitating greater access to evidence-based psychological therapies (Knapstad, Sæther, Hensing, & Smith, 2020; Muñoz-Navarro et al., 2024). In England, the National Health Service (NHS) expanded the NHS Talking Therapies (NHSTT, formerly known as Improving Access to Psychological Therapies) as part of the NHS Long-Term Plan (NHS, 2019). In addition to improving CMD symptoms, the Long-Term Plan seeks to improve access to mental health support for people in work, and to support those seeking work and retaining employment (NHS England, 2016b). Increasing productivity was an anticipated byproduct of improving mental health symptoms and was part of the initial plan for NHSTT (Clark, 2018). Several studies have provided evidence that psychological treatments delivered by services like NHSTT are associated with improved employment outcomes shortly after discharge, as well as reduced healthcare utilization (Clark et al., 2009; Gruber et al., 2022; Layard, 2017; Toffolutti et al., 2021; Wakefield et al., 2021). However, there is limited evidence around whether psychological therapies lead to sustained long-term improvements in employment outcomes. Evidence from Norway’s PRomPT service (based on NHSTT) shows increases in personal income and rates of working without receiving state benefits during the 5 years posttreatment compared to treatment as usual in primary care (Smith et al., 2025). In Spain, the PSicAP trial evaluating the impact of transdiagnostic cognitive behavioral therapy (TCBT) versus routine primary care demonstrated that individuals receiving TCBT experienced a significant improvement in personal income in the year after treatment (Muñoz-Navarro et al., 2024). Conversely, evidence from Denmark from a service similar to NHSTT found no effect on labor market outcomes (Serena, 2025).

To our knowledge, there are no England-based studies that evaluate the long-term labor market effects of NHSTT using national population data on individual-level payment records. Having such estimates would be crucial to provide inputs for cost-benefit calculations. Additionally, it will provide evidence supporting the increased spending in NHSTT committed by the UK government to get people with CMDs back into work (UK Government, 2023). While the NHSTT has been found to be cost-effective – with every pound spent, generating an additional benefit of £5.50 for the economy – these calculations are based on extrapolating the short-term effects of therapy (Oparina, Krekel, & Srisuma, 2024).

In this study, we first aim to evaluate the effects of NHSTT treatment completion on labor market outcomes such as employment and earnings. Second, we evaluate whether the effect of treatment completion on labor market outcomes varies across different sociodemographic and treatment-related characteristics.

## Methods

### Study design

Using a unique data linkage between the 2011 Census in England and Wales, His Majesty’s Revenue and Customs’ (HMRC’s) Pay As You Earn (PAYE) records, Office for National Statistics (ONS) death registrations and the NHSTT national dataset, we conducted a retrospective, quasi-experimental panel-data study evaluating the average treatment effect of psychological therapies on probability of being a paid employee and monthly employee earnings. Ethical approval was obtained from the National Statistician’s Data Ethics Advisory Committee (NSDEC23(18)).

### Data sources

The 2011 Census is a decennial population-based survey conducted by the ONS to collect information on people and households in England and Wales (response rate = 93.9%) (Office for National Statistics, 2016). The NHSTT program is a primary mental health service in England, which offers evidence-based psychological therapies for people with CMDs (Clark, 2018). All services across England collect standardized measures contributing toward the NHSTT Minimum Data Set. These data are processed into monthly and annual submissions by NHS England (John et al., 2022; National Collaborating Centre for Mental Health, 2021). We obtained annual adult NHSTT data from NHS England via the Data Access Request Service.

Labor market outcomes were derived from the HMRC PAYE dataset, which is collected for tax purposes in the United Kingdom. This dataset stores information submitted by employers on their employees’ salaries, bonuses, payrolled expenses, redundancy payments or allowances (Office for National Statistics, 2024). The dataset covers the whole employee population in the United Kingdom, excluding people who are self-employed or receiving income from other sources such as investments or property rentals. Supplementary Table 1 provides a list of data sources and time periods.

### Data linkage

NHSTT records were linked to the 2011 Census and death registrations via NHS number. The 2011 Census was linked to the 2011–2013 Patient Registers to retrieve NHS numbers for census respondents, with a linkage rate of 95.3% (Nafilyan et al., 2024).

The HMRC PAYE data between 1 April 2014 and 31 December 2022 was linked via the encrypted National Insurance (NI) number. To obtain NI numbers, the census records were indexed to the ONS Demographic Index data (Archer et al., 2022) via NHS number, with 89.7% of census records successfully linked to NI numbers.

### Data structure

HMRC PAYE payments were calendarized into monthly records for individuals, then aggregated into quarters to reduce the dataset size. Where an individual had multiple quarterly payments (e.g. due to multiple employments), the recorded pay was summed across all matching records for each quarter. Our final linked dataset had a panel structure with quarterly records for individuals. The quarters were defined using calendar months: January–March (Q1), April–June (Q2), July–September (Q3), and October–December (Q4).

### Study population

Our study population includes individuals with an NHSTT referral from 1 April 2016 to 31 March 2020, who attended at least one therapy session (that was not an assessment) and were scoring in the clinical range for either depression or an anxiety disorder (NHS England, 2024b) at referral. Participants were included in the sample when the reason for the termination of the treatment (as reported by the therapist) was one of the following: (1) the patient completed scheduled treatment (exposed group); (2) suitable for NHSTT, but the patient refused the treatment that was offered (nonexposed group); or (3) the patient dropped out of treatment (unscheduled discontinuation; nonexposed group) (Supplementary Table 2). Participants must also have been enumerated in 2011 Census and have valid NHS and NI numbers. The population was restricted to those aged 25–60 years at the time of referral and living in England. This age restriction was implemented as younger adults (18–24 years) are likely to be in full-time education or moving into employment; therefore, any effect may be driven by natural movement into the workforce. In selecting 25 years as a minimum age, we had a fuller history of labor market activity for all individuals within the analysis. We removed participants who died in the same calendar quarter as the first therapy session and those who reported they were retired at the time of referral into NHSTT. Participants who died later during the follow-up period were retained in the study population, but follow-up was censored at the time of death. For participants with multiple referrals, we selected the earliest one.

### Exposure variable

The exposed group was those who completed treatment. The nonexposed group comprised participants who dropped out of treatment.

The main exposure was the time since the first therapy (excluding the initial assessment) for people who completed the treatment. Time period 0 refers to the date (year and quarter) in which the first therapy occurred; all modeled estimates of changes in pay and the likelihood of employment are relative to this reference period.

### Outcome variables

Two outcomes were analyzed: monthly employee pay and paid employee status (binary). Monthly employee pay was derived by dividing the gross quarterly pay by three. Monthly pay was imputed to be 0 if it was negative, winsorized at the 99.9% centile to remove erroneously large values, and deflated to 2023 prices using the Consumer Price Index including owner occupiers’ housing costs. Being a paid employee was defined as receiving any monthly pay > 0. Participants who had an NI number but no records in the HMRC PAYE dataset (e.g. due to unemployment, long-term sickness, or self-employment) were included in the analysis, with their earnings set to 0.

### Covariates

Sociodemographic characteristics, including age (calculated based on date of birth measured in quarter-year), country of birth, number of dependent children, relative area deprivation, disability, ethnicity, highest qualifications, National Statistics Socio-Economic Classification (NS-SEC), region, and sex, were derived from the 2011 Census (Supplementary Table 3). The date of death was derived from death registrations.

Diagnosis, employment status, psychotropic medication use, number of treatment sessions, source of referral, therapy intensity, and recovery types were taken from NHSTT data. Self-reported employment status and medication use are recorded at the beginning of therapy. Recovery types (reliable recovery, reliable improvement, reliable deterioration, and no change) were predefined by NHSTT and are based on scores from the Patient Health Questionnaire-9 and Generalized Anxiety Disorder-7 (or Anxiety Disorder Specific Measure) taken at the beginning and end of therapy (Clark, 2018). We combined these into (1) reliable recovery or reliable improvement, and (2) reliable deterioration or no change.

### Statistical analysis

Covariate balance between exposure groups was assessed using absolute standardized differences, with values <10% indicating acceptable balance (Normand et al., 2001). To create a nonexposed group that was similar to the exposed group in terms of observed characteristics, we fitted a logistic regression including characteristics exhibiting the largest between-group imbalance to estimate the probability – or propensity – of completing treatment. Weights for everyone were calculated as the inverse of the probability of completing treatment; 1/propensity score for the exposed and 1/(1-propensity score) for the nonexposed. These inverse probability weights (IPWs) were then incorporated into the fixed-effects regression models. Including IPWs in the model helped balance the covariate distribution across exposure groups, improving the estimation of the effects of aging and calendar time (independently of the treatment effect) and mitigating against pretreatment trends in outcomes. The variables with a >10% difference were: age band, number of dependent children, relative area deprivation, employment status, highest qualification, NS-SEC, and region. Disability was not included despite the standardized difference being 12.1%, as the inclusion of this variable made little difference to the final results. We excluded recovery types and the number of treatment sessions as they are posttreatment variables and, therefore, may be directly correlated with the outcome measures.

We used an event study approach, the estimation equation (equation (1)) modeled the outcomes for individual *i* observed at time *t* (measured in quarter-years) and was fitted using linear regression with individual fixed effects ( 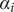 ) to capture time-invariant confounding factors. Fixed effects for each year since the first therapy ( 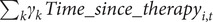 ), from 4 years before the start to 7 years after the start, were included to capture natural recovery. The exposure of interest was the interaction between time since the first therapy session and a time-invariant indicator variable for having completed the treatment, with time 0 being excluded. The models were adjusted for quarterly age and calendar time (quarter and year) to capture age-related employment progression and changes in macroeconomic conditions. Participants’ follow-up was censored at the earliest of turning 64 years old, death, or the end of the study (31 December 2022). Pre-therapy follow-up was censored when participants were aged <21 years.(1)
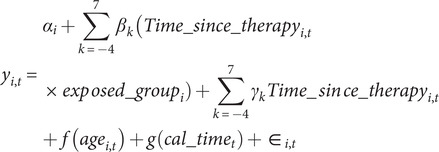

All reported confidence intervals around point estimates are at the 95% level and are estimated using robust standard errors clustered at an individual level to capture any intra-person correlation between observations. Statistical significance was determined by *p* < 0.05. All reported estimates are derived from regression models that incorporate IPWs. Analysis of labor market outcomes was stratified by age band, deprivation, diagnoses, employment status at referral, ethnicity, highest qualifications, psychotropic medication usage, NS-SEC, region, sex, and therapy intensity.

The initial processing of HMRC PAYE data was performed in Python version 3.6.8. Further processing and analysis were conducted in R version 4.1.3. All data were de-identified before being analyzed in the secure data environment at the ONS.

We complement the traditional subgroup analysis with a data-driven machine-learning approach to identify other key sources of heterogeneity, using a Generalized Random Forest approach (Athey, Tibshirani, & Wager, 2019) (Supplementary Appendix A). We conducted several sensitivity analyses looking at (1) effects of reliable recovery and improvement, (2) referred but not treated as a new control group, and (3) the inclusion of younger adults (18–21 years) in the sample (Supplementary Appendix B).

## Results

### Patient demographics

**Figure 1.**
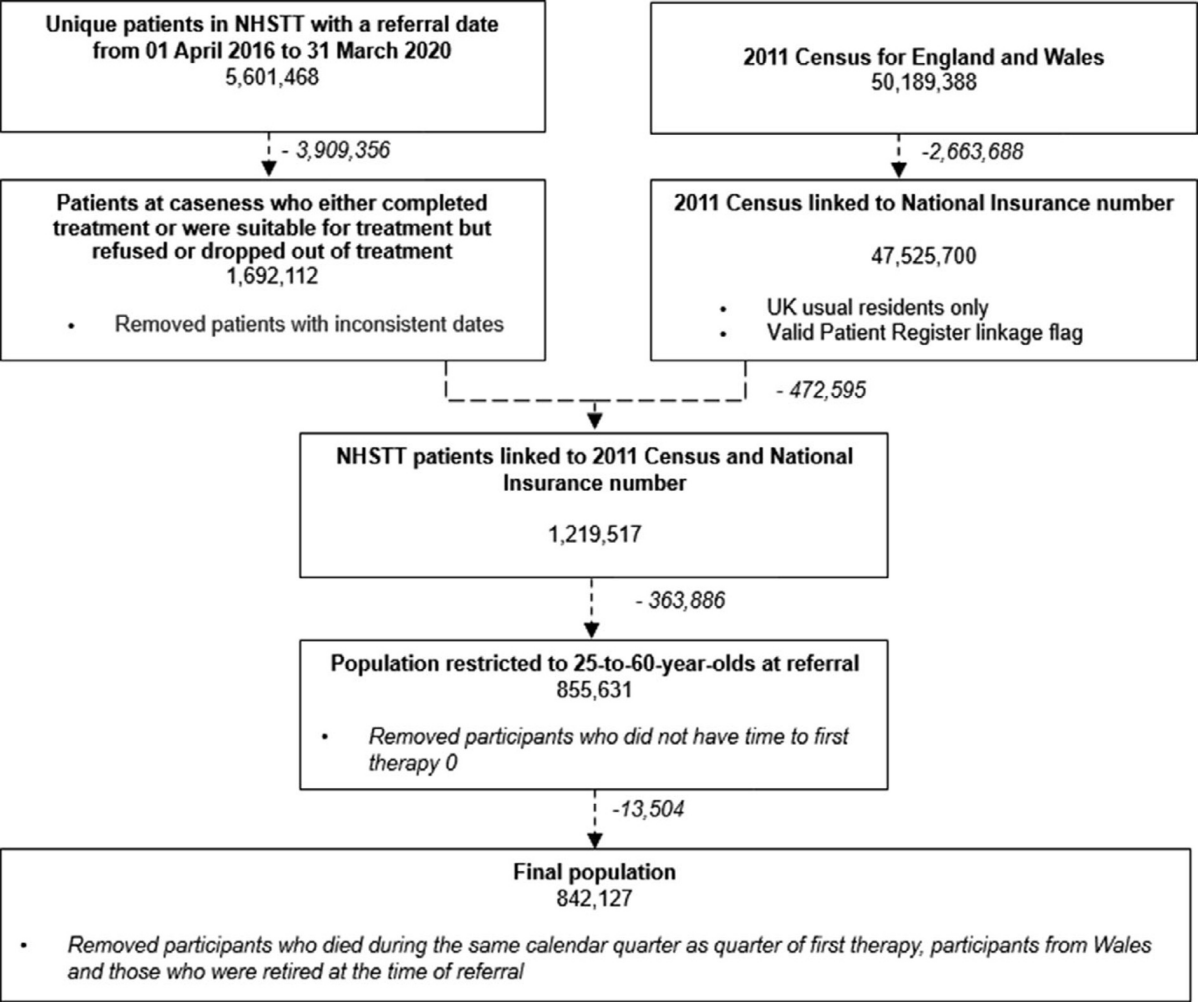
Population and linkage flow diagram. *Notes*: (1) Caseness is defined by patients scoring in the clinical range for either depression or an anxiety disorder. (2) The Patient Register flag indicates whether an individual can be linked to the 2011–2013 Patient Registers to retrieve NHS numbers for census respondents.

Of the 842,127 participants (mean age 40.5 years, standard deviation = 10.1) included in the analysis (Figure 1), 593,300 (70.5%) were rated by their therapist to have completed treatment during the study period, and 248,827 (29.5%) were considered as having dropped out of treatment. The study population predominantly comprised female (66.6% for the exposed group and 67.8% for the nonexposed group), from a white ethnic background (90.2% and 89.7%, respectively), who self-referred into NHSTT (74.1% and 72.6%, respectively) (Table 1) and with primary diagnosis of depression (38.0% and 38.9%, respectively) or GAD (24.5% and 23.2%, respectively) (Table 2). The average number of therapy sessions for the exposed group was 8.3 compared to 4.5 for the nonexposed group.

**Table 1.** Sociodemographic summary statistics between the completed and dropped out groups, including the absolute standardized difference test

Characteristic	Category	Completed treatment (%)	Dropped out of treatment (%)	All participants (%)	ASD^a^
Age band	25–34	192,190 (32.4%)	98,525 (39.6%)	290,715 (34.5%)	14.3%
	35–44	167,783 (28.3%)	70,893 (28.5%)	238,676 (28.3%)	
	45–54	160,095 (27.0%)	56,665 (22.8%)	216,760 (25.7%)	
	55–60	73,232 (12.3%)	22,744 (9.1%)	95,976 (11.4%)	
Country of birth	Non-UK	55,321 (9.3%)	21,484 (8.6%)	76,805 (9.1%)	2.4%
	UK	537,979 (90.7%)	227,343 (91.4%)	765,322 (90.9%)	
Dependent children	No children	310,092 (52.3%)	118,910 (47.8%)	429,002 (50.9%)	19.3%
	One child	131,479 (22.2%)	59,805 (24.0%)	191,284 (22.7%)	
	Two children	104,058 (17.5%)	44,613 (17.9%)	148,671 (17.7%)	
	Three or more children	40,431 (6.8%)	22,628 (9.1%)	63,059 (7.5%)	
	Missing or not stated	7,240 (1.2%)	2,871 (1.2%)	10,111 (1.2%)	
Deprivation^b^	1 dimension	169,134 (28.5%)	75,307 (30.3%)	244,441 (29%)	30.0%
	2 dimensions	76,815 (12.9%)	41,702 (16.8%)	118,517 (14.1%)	
	3 dimensions	25,614 (4.3%)	17,014 (6.8%)	42,628 (5.1%)	
	4 dimensions	2,474 (0.4%)	1,845 (0.7%)	4,319 (0.5%)	
	Not deprived	312,023 (52.6%)	110,088 (44.2%)	422,111 (50.1%)	
	Missing or not stated	7,240 (1.2%)	2,871 (1.2%)	10,111 (1.2%)	
Disability	Activities limited a lot	32,937 (5.6%)	16,931 (6.8%)	49,868 (5.9%)	12.1%
	Activities limited a little	51,503 (8.7%)	23,581 (9.5%)	75,084 (8.9%)	
	Not disabled or activities not limited	508,860 (85.8%)	208,315 (83.7%)	717,175 (85.2%)	
Employment status	Employed	404,137 (68.1%)	148,368 (59.6%)	552,505 (65.6%)	15.3%
	Not working, long-term sick/disabled	40,554 (6.8%)	22,667 (9.1%)	63,221 (7.5%)	
	Student	6,180 (1.0%)	3,148 (1.3%)	9,328 (1.1%)	
	Not working, not seeking work	43,752 (7.4%)	21,380 (8.6%)	65,132 (7.7%)	
	Not working, seeking work	42,300 (7.1%)	27,143 (10.9%)	69,443 (8.2%)	
	Missing or not stated	56,377 (9.5%)	26,121 (10.5%)	82,498 (9.8%)	
Ethnicity	Asian	27,726 (4.7%)	11,727 (4.7%)	39,453 (4.7%)	5.0%
	Black	14,408 (2.4%)	6,491 (2.6%)	20,899 (2.5%)	
	Mixed	11,953 (2.0%)	5,695 (2.3%)	17,648 (2.1%)	
	Other	3,916 (0.7%)	1,727 (0.7%)	5,643 (0.7%)	
	White	535,297 (90.2%)	223,187 (89.7%)	758,484 (90.1%)	
Highest qualification	Apprenticeship	10,326 (1.7%)	4,641 (1.9%)	14,967 (1.8%)	37.2%
	Level 1	87,834 (14.8%)	46,662 (18.8%)	134,496 (16%)	
	Level 2	115,452 (19.5%)	54,337 (21.8%)	169,789 (20.2%)	
	Level 3	117,502 (19.8%)	45,529 (18.3%)	163,031 (19.4%)	
	Level 4 and above	202,720 (34.2%)	59,191 (23.8%)	261,911 (31.1%)	
	No qualifications	43,646 (7.4%)	31,200 (12.5%)	74,846 (8.9%)	
	Other	15,820 (2.7%)	7,267 (2.9%)	23,087 (2.7%)	
NS-SEC^c^	Full-time students	57,127 (9.6%)	24,685 (9.9%)	81,812 (9.7%)	28.1%
	Higher managerial, administrative, and professional	53,056 (8.9%)	14,571 (5.9%)	67,627 (8.0%)	
	Intermediate occupations	103,738 (17.5%)	39,008 (15.7%)	142,746 (17.0%)	
	Long-term unemployed	12,533 (2.1%)	8,640 (3.5%)	21,173 (2.5%)	
	Lower managerial, administrative, and professional	145,248 (24.5%)	47,793 (19.2%)	193,041 (22.9%)	
	Lower supervisory and technical occupations	33,157 (5.6%)	14,757 (5.9%)	47,914 (5.7%)	
	Never worked	16,970 (2.9%)	12,870 (5.2%)	29,840 (3.5%)	
	Routine occupations	48,615 (8.2%)	26,466 (10.6%)	75,081 (8.9%)	
	Semi-routine occupations	91,325 (15.4%)	46,666 (18.8%)	137,991 (16.4%)	
	Small employers and own-account workers	31,531 (5.3%)	13,371 (5.4%)	44,902 (5.3%)	
Region	East Midlands	66,959 (11.3%)	18,866 (7.6%)	85,825 (10.2%)	18.4%
	East of England	57,581 (9.7%)	27,039 (10.9%)	84,620 (10%)	
	London	79,800 (13.5%)	35,639 (14.3%)	115,439 (13.7%)	
	North East	39,944 (6.7%)	17,926 (7.2%)	57,870 (6.9%)	
	North West	77,891 (13.1%)	39,860 (16.0%)	117,751 (14.0%)	
	South East	106,104 (17.9%)	33,290 (13.4%)	139,394 (16.6%)	
	South West	53,307 (9.0%)	25,220 (10.1%)	78,527 (9.3%)	
	West Midlands	53,556 (9.0%)	22,455 (9.0%)	76,011 (9.0%)	
	Yorkshire and The Humber	58,158 (9.8%)	28,532 (11.5%)	86,690 (10.3%)	
Sex	Females	395,041 (66.6%)	168,693 (67.8%)	563,734 (66.9%)	2.6%
	Males	198,259 (33.4%)	80,134 (32.2%)	278,393 (33.1%)	

a Absolute standardized differences (ASDs) are calculated to assess covariate balance between the completed treatment group and the dropped out of treatment group at baseline.

b Deprivation = The dimensions of deprivation used to classify households are indicators based on four selected household characteristics: education, employment, health, and housing.

c NS-SEC, National Statistics Socio-Economic Classification.

**Table 2.** Mental health and treatment summary statistics between the completed and dropped out groups, including the absolute standardized difference test

Characteristic	Category	Completed treatment (%)	Dropped out of treatment (%)	All participants (%)	ASD^a^
Diagnosis	Agoraphobia	3,166 (0.5%)	2,161 (0.9%)	5,327 (0.6%)	8.0%
	Depression	225,339 (38.0%)	96,887 (38.9%)	322,226 (38.3%)	
	Generalized anxiety disorder	145,633 (24.5%)	57,659 (23.2%)	203,292 (24.1%)	
	Mixed anxiety and depressive disorder	60,874 (10.3%)	24,298 (9.8%)	85,172 (10.1%)	
	Obsessive-compulsive disorder	12,774 (2.2%)	4,526 (1.8%)	17,300 (2.1%)	
	Other anxiety or stress-related disorder	26,092 (4.4%)	9,261 (3.7%)	35,353 (4.2%)	
	Panic disorder	14,611 (2.5%)	6,983 (2.8%)	21,594 (2.6%)	
	Post-traumatic stress disorder	22,062 (3.7%)	10,033 (4.0%)	32,095 (3.8%)	
	Social phobias	11,805 (2.0%)	5,451 (2.2%)	17,256 (2.0%)	
	Specific (isolated) phobias	4,355 (0.7%)	1,556 (0.6%)	5,911 (0.7%)	
	Missing or not stated	66,589 (11.2%)	30,012 (12.1%)	96,601 (11.5%)	
Psychotropic Medication	Not prescribed	253,962 (42.8%)	91,263 (36.7%)	345,225 (41%)	38.5%
	Prescribed, not taking	25,601 (4.3%)	12,754 (5.1%)	38,355 (4.6%)	
	Prescribed, taking	284,327 (47.9%)	132,358 (53.2%)	416,685 (49.5%)	
	Missing or not stated	29,410 (5.0%)	12,452 (5.0%)	41,862 (5.0%)	
Number of treatment sessions	2	28,387 (4.8%)	72,414 (29.1%)	100,801 (12.0%)	111.3%
	3–5	154,457 (26.0%)	115,087 (46.3%)	269,544 (32.0%)	
	6–8	206,771 (34.9%)	38,390 (15.4%)	245,161 (29.1%)	
	9–15	149,332 (25.2%)	19,228 (7.7%)	168,560 (20.0%)	
	16 or more	54,353 (9.2%)	3,708 (1.5%)	58,061 (6.9%)	
Source of referral	Other	34,729 (5.9%)	16,466 (6.6%)	51,195 (6.1%)	14.3%
	Primary health care	116,870 (19.7%)	51,233 (20.6%)	168,103 (20.0%)	
	Self-referral	439,684 (74.1%)	180,624 (72.6%)	620,308 (73.7%)	
	Missing or not stated	2,017 (0.3%)	504 (0.2%)	2,521 (0.3%)	
Therapy intensity	High intensity	397,578 (67.0%)	141,379 (56.8%)	538,957 (64.0%)	21.1%
	Low intensity	193,951 (32.7%)	106,591 (42.8%)	300,542 (35.7%)	
	Missing or not stated	1,771 (0.3%)	857 (0.3%)	2,628 (0.3%)	
Recovery types	Deterioration	14,929 (2.5%)	22,918 (9.2%)	37,847 (4.5%)	201.1%
	Improvement	94,193 (15.9%)	79,518 (32.0%)	173,711 (20.6%)	
	No change	58,830 (9.9%)	95,069 (38.2%)	153,899 (18.3%)	
	Recovery	424,142 (71.5%)	47,528 (19.1%)	471,670 (56.0%)	
	Missing or not stated	1,206 (0.2%)	3,794 (1.5%)	5,000 (0.6%)	

a Absolute standardized differences (ASDs) are calculated to assess covariate balance between the completed treatment group and the dropped out of treatment group at baseline.

For both exposure groups, the mean follow-up was 13.7 calendar quarters before therapy, while the mean follow-up after the first therapy was 18.7 quarters for the exposed group and 18.6 quarters for the nonexposed group (Supplementary Table 4). On the date of the first therapy (quarter-year), 68.7% of individuals were in paid employment (70.9% for exposed and 63.4% for nonexposed), while the mean monthly deflated earnings was £1,615.0 (£1,716.6 exposed and £1,372.9 nonexposed) when including those not in work, and £2,351.79 (£2,421.6 exposed and £2,165.6 nonexposed) when excluding those not in work (Supplementary Table 5). Before adjustments, the exposed group earned more on average than the nonexposed group before and after treatment; the IPWs made the earnings trajectories more parallel (Supplementary Figure 1 and see Supplementary Figure 2 for balance plot).

### Effect of treatment completion on monthly earnings and employee status

**Figure 2.**
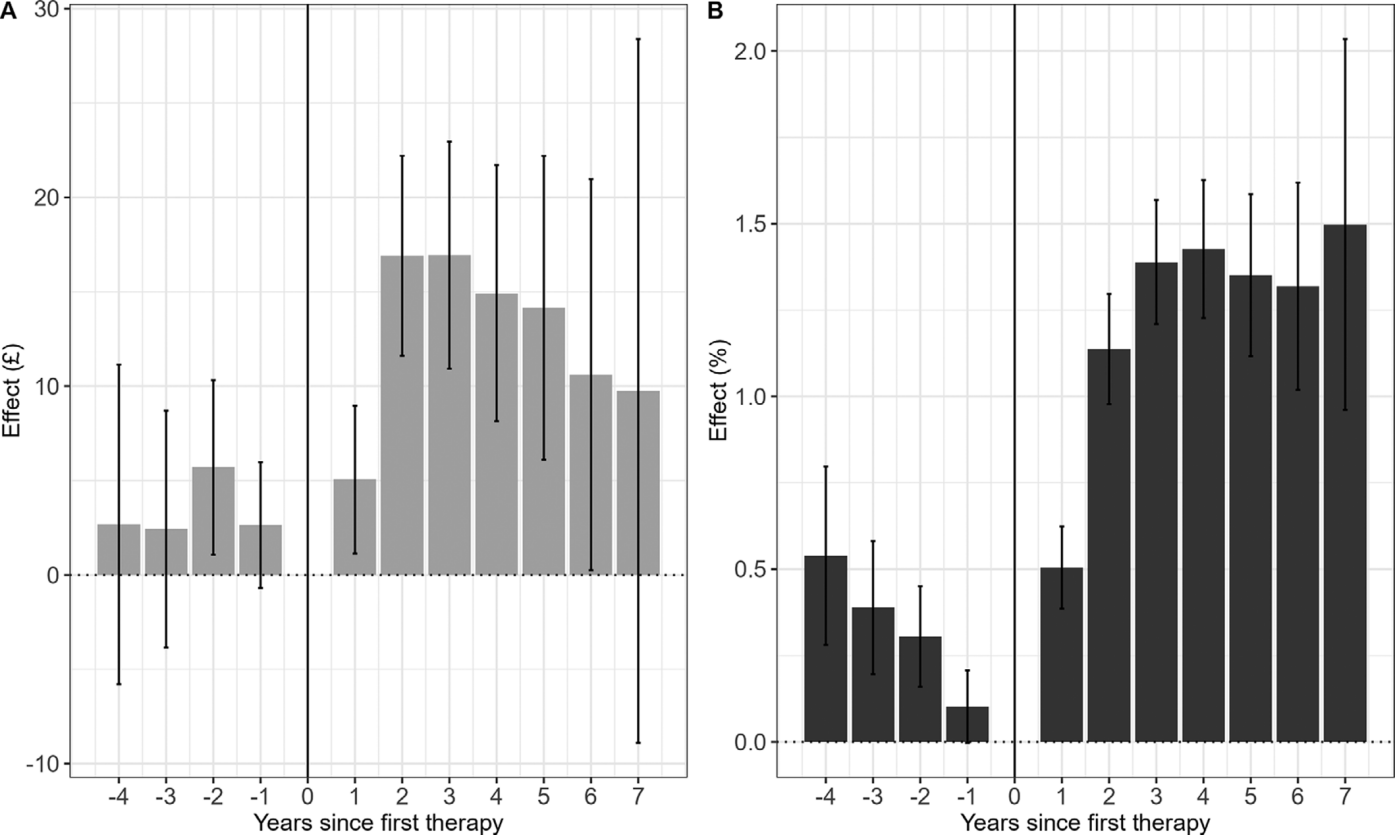
Effects of NHSTT treatment completion on (A) monthly earnings and (B) probability of being a paid employee. *Notes*: (1) All reported confidence intervals around point estimates are at the 95% level and are estimated using robust standard errors clustered at an individual level. (2) Estimates were derived from models that incorporated Inverse Probability Weights.

The effect of NHSTT on monthly earnings reached a maximum of £16.9 more per month (95% confidence interval [CI]: 11.6–22.2) in year 2 after treatment, and the statistically significant increase relative to pretreatment was sustained at £10.6 (95% CI: 0.2–21.0) 6 years after treatment. Though the magnitude of the effect remained similar in year 7 to year 6, the results were no longer statistically significant due to diminished power (Figure 2). We also found statistically significant evidence that completing NHSTT is associated with an increase in the probability of being a paid employee; the maximum effect reached and was sustained at 1.5 p.p. (95% CI: 1.0–2.0) 7 years after the first therapy (Figure 2). Estimates were derived from models that incorporated IPWs.

### Heterogeneous treatment effects by sociodemographic characteristics

**Figure 3.**
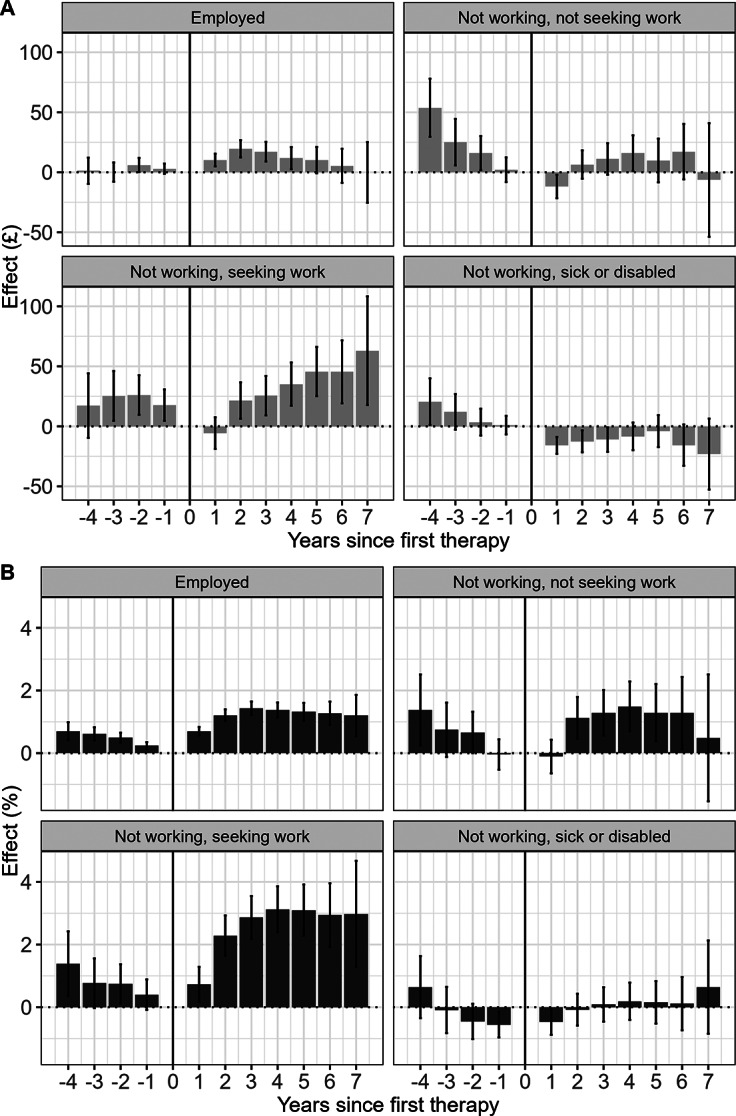
Effects of NHSTT treatment completion on (A) monthly earnings and (B) probability of being a paid employee broken down by self-reported employment status. *Notes*: (1) All reported confidence intervals around point estimates are at the 95% level and are estimated using robust standard errors clustered at an individual level. (2) Estimates were derived from models that incorporated Inverse Probability Weights. (3) Self-reported employment status is recorded at the beginning of therapy.

Participants who reported being ‘Not working, seeking work’ at the beginning of therapy saw the greatest increases in average monthly earnings and the probability of being employed; compared with the 1 year before first therapy, the effect on monthly earnings reached a maximum of £63.0 (95% CI: 17.7–108.3) more per month by year 7 after treatment. Probability of employment reached a maximum of 3.1 p.p. (95% CI: 2.4–3.9) in year 4, and the increase was sustained at 3.0 p.p. (95% CI: 1.3–4.7) by year 7 (Figure 3). The effect size on the probability of being a paid employee was smaller but still positive for the ‘Not working, not seeking work’; 1.5 p.p. (95% CI: 0.7–2.3) 4 years after therapy (largest year effect) and 0.5 p.p. (95% CI: -1.5 to -2.5) in year 7 (there was no statistically significant effect on earnings in this group).

For both females and males, monthly earnings were highest in year 3 after treatment (£15.9 [95% CI: 9.3–22.5] for females and £24.8 [95% CI: 12.4–37.2] for males). The probability of being a paid employee was highest in year 7 after treatment; compared with the year of first therapy, the effect reached 1.4 p.p. (95% CI: 0.5–2.3) for males and 1.5 p.p. (95% CI: 0.9–2.2) for females (Supplementary Table 7).

Overall, completing NHSTT treatment had the largest effect in the age groups 25–34 and 35–44 years; the effect reached maximum of 2.3 p.p. (95% CI: 1.4–3.2) for the 25- to 34-year-olds (in year 7) and 2.0 p.p. (95% CI: 1.6–2.4) for the 35- to 44-year-olds (in year 5) (Supplementary Table 7). Results for 45- to 54-year-olds were smaller but still positive, with the highest probability reaching 1.0 p.p. (95% CI: 0.6–1.3) in year 3. By year 7 after treatment, the increase relative to pretreatment was sustained at 1.9 p.p. (95% CI: 0.9–2.8) for 35- to 44-year-olds, and 1.0 p.p. (95% CI: 0.0–2.0) for 45- to 54-year-olds. Completing NHSTT treatment had no statistically significant effect on individuals aged 55–60 years. See Supplementary Tables 6 and 7 for further sociodemographic and treatment characteristic breakdowns and impact on monthly earnings.

## Discussion

This paper is the first nationally representative analysis of long-term labor market effects of psychological therapies on employment and earnings globally. Our findings show that completing NHSTT is associated with a sustained improvement in labor market outcomes: a 1.5 p.p. increase in the probability of being a paid employee 7 years after treatment and a £16.9 average increase in monthly earnings 2 years after treatment. In 2023/24, 1.8 million people were referred to NHSTT, of whom 671,648 completed treatment (NHS England, 2024a); therefore, a 1.5 p.p increase in employment equates to around 10,000 people annually.

Research has identified that CMDs are associated with an increased risk of economic inactivity and reduced wages, with some studies suggesting a potential causal link (Andersen, Richmond-Rakerd, Moffitt, & Caspi, 2024; Frijters, Johnston, & Shields, 2014; Germinario, Amin, Flores, & Flores-Lagunes, 2022). These studies highlight the importance of psychological treatment for getting people into, or retaining them in, employment and increasing earnings. Previous research has identified short-term benefits of NHSTT treatment on labor market outcomes, suggesting between 4 and 10 percentage point increase in the employment rate over a maximum follow-up of 72 weeks (Clark et al., 2009; Toffolutti et al., 2021). These studies used self-report methods, which are potentially subject to respondent bias, without suitable nonexposed groups for comparison (Clark et al., 2009; Toffolutti et al., 2021). Our research supports these initial studies, confirming positive labor market outcomes after NHSTT treatment. While we see smaller overall improvements in the likelihood of being employed, we demonstrate that these increases are sustained long term. We are also confident that these increases are due to completing treatment because of our within-individual estimation approach, and the comparison to the nonexposed group of people who interrupted treatment earlier than expected.

In the pretreatment period, the treatment group had higher employment and earnings than the control group (Figures 2–3), but these advantages declined before treatment, which likely reflects the timing of starting treatment in response to mental health deterioration. Psychological treatment may be administered at a time when individuals have low income, due to an inability to work resulting from mental ill health, prompting referrals to NHSTT. While some of the observed posttreatment increase may be attributed to “regression to the mean”, that is, natural recovery following an acute illness irrespective of treatment, the posttreatment effect surpasses the observed pretreatment peak. This indicates the presence of additional influencing factors. Furthermore, our findings are in line with research from low- and middle-income countries on the effects of mental health interventions on the labor market (Lund et al., 2024) supporting the existence of a genuine treatment effect. Official statistics show that a larger number of people aged 16–34 years are inactive in the labor market due to depression and anxiety (Office for National Statistics, 2022b). We find the probability of being a paid employee following NHSTT treatment to be highest in younger people, highlighting that targeting treatment for the groups mostly affected by CMDs (NHS England, 2016a) can improve the economic health of the country long term. While the probability of being a paid employee showed strong results for the youngest age group, the estimated effect on monthly earnings was on an increasing trajectory during the pretreatment period, making it challenging to confidently conclude that treatment is associated with the total increased pay in this age group.

We found no significant findings for individuals aged 55–60 years. While previous evaluations of psychological therapies found older people tend to respond better to treatment and reach higher recovery rates than other age groups (Saunders et al., 2021), they do not appear to have improved long-term employment outcomes. This might be due to a greater proportion of older adults taking on unpaid caregiving responsibilities (Carers UK, 2024).

Individuals who were not working but seeking work at the time of referral benefitted the most from completing NHSTT treatment. This characteristic was also identified as a meaningful contributor to heterogeneity in probability of being a paid employee. This suggests that the relationship between NHSTT and labor market outcomes is driven by individuals moving into employment, supporting the idea that NHSTT can facilitate the unemployed to take up new jobs. We observed a downward trend in monthly earnings among the employed population. One plausible explanation, as suggested by Virtanen et al. (2011) and Voglino et al. (2022), is that individuals might reduce their working hours or move to lower-paid roles to facilitate improvements in mental health.

### Strengths and limitations

This study used a population-level linked dataset, which enabled us to analyze heterogeneous treatment effects and understand not only the average effect of treatment, but also which patients benefit the most or the least. These findings are important for both equity considerations and program targeting. Another strength of our study is the use of fixed-effects (within-person) regression modeling, which implicitly controls for time-invariant confounders that do not change over time, irrespective of whether they are measured and observable. However, the model only controls for quarterly age and calendar time and does not control for other cofounders that change over time. Unmeasured confounders that might affect an individual’s earnings, such as bereavement, lack of job opportunities, or changes in individuals’ personal and family circumstances, cannot be controlled for. Future research should aim to account for these.

NHSTT does not provide a pre-planned schedule for the course of treatment for each patient; therefore, the definition of completed treatment is based on a clinician-defined reason for the end of the care episode. There is a risk that if therapists differ in how they define or record treatment completion, the variable may not consistently reflect actual treatment completion. This could bias estimates of treatment effectiveness. There is potential that if therapists are more likely to report completion based on the number of attended sessions, or for patients who showed improvement, the variable could be endogenously related to outcome. In addition, patients who dropped out might have done so because they recovered with a smaller dose of therapy. Some therapeutic exposure in the nonexposed group could lead to an underestimation of the magnitude of the effect in this study. We did not have access to the initial screening questionnaire scores to control for severity in our model. However, we ensured that all participants in our study were scored within the clinical range for depression and/or anxiety as flagged by NHS England. Additionally, we included therapy intensity in the IPW model to account for variations in treatment.

The HMRC PAYE dataset is limited to people in paid employment, excluding ~4.4 million individuals who are self-employed (12–15% of total employment, Office for National Statistics, 2025). This exclusion introduces measurement error, as those who are self-employed are classified as out of work. If psychological therapy treatment increases the probability of being self-employed, in the same way as it does for employment, our results would underestimate the effect of psychological therapy on total earnings. However, in 2022, the number of payrolled employees reached 29.9 million (Office for National Statistics, 2024), suggesting our results are highly relevant for the majority of the working population.

Additionally, self-employment is more prevalent among older age groups (Office for National Statistics, 2023a) and, given that the present sample excludes individuals aged under 25, data may be disproportionately missing in our cohort. Older participants may appear to have a lower treatment effect simply because a larger proportion is not in PAYE, and not because they earn less.

While younger adults are a key demographic in NHSTT (Barker & Kirk-Wade, 2024), excluding younger adults from this analysis may not fully represent the NHSTT population. However, the effects seen for that age group may be driven by natural movement into the workforce, suggesting the effects may reflect broader life transitions rather than isolated psychological therapy effects.

A potentially larger threat to the identification strategy is that the decision to discontinue treatment is endogenous, meaning those who dropped out might systematically differ from those who remained in the treatment, which may have affected the estimation of the effects of aging and calendar time. The IPW addresses this in the best available way; however, we can only control for observable characteristics.

Lower income or probability of employment could be a positive outcome for an individual, especially if work is the direct cause of mental health issues (e.g. due to being in an adverse psychosocial environment or working long hours). Future research should investigate other labor market outcomes, such as sustained employment or receipt of social security benefits.

Lastly, our study is a nonrandomized comparison. It has the advantage that it uses complete population data, but we may have underestimated labor market effects due to some level of exposure in both groups. Consistent with this, the observed positive associations between psychological therapies and labor market outcomes are less than half of those observed in the randomized controlled trial of the PRomPT service (Smith et al., 2025). Taken together, our study and the smaller randomized comparison make a compelling case for the beneficial effects of NHSTT-style services on labor market participation.

## Conclusions

Completion of psychological treatment for CMDs through the national NHSTT program leads to sustained increases in both employment and earnings up to 7 years after the start of treatment, equating to ~10,000 people back into employment annually (NHS England, 2024a). Policymakers may consider ways to invest in services such as NHSTT to reduce the impact of CMDs on the economy. Future research should explore a broader range of outcomes, such as social security benefit receipts, and consider the implications of the employment advisors’ service provided by NHSTT to understand in more detail the mechanisms underpinning our findings.

## Supporting information

10.1017/S003329172510305X.sm001 Rzepnicka et al. supplementary material Rzepnicka et al. supplementary material

## Data Availability

The source data are not publicly available and are subject to controlled access due to their sensitive nature. Non-linked death registrations data are available to Accredited Researchers through the Secure Research Service (SRS). Details of the application requirements and process, and the use of data, are available at https://www.ons.gov.uk/aboutus/whatwedo/statistics/requestingstatistics/secureresearchservice. The NHS Talking Therapies dataset is held by NHS England and can be accessed through the NHS Secure Data Environment: https://digital.nhs.uk/services/secure-data-environment-service.

## Supplementary material

The supplementary material for this article can be found at http://doi.org/10.1017/S003329172510305X.
