# Supplementary material for: The effect of adult psychological therapies on employment and earnings: Evidence from England
Source: Psychol Med. 2026 Feb 27;56:e33. doi: 10.1017/S003329172510305X (PMC12973244; doi:10.1017/S003329172510305X)
Supplement: Rzepnicka et al. supplementary material [file S003329172510305Xsup001.docx]

**Supplementary material**

[**Supplementary Table 1.** Data sources and time periods covered 2](#_Toc214978789)

[**Supplementary Table 2.** Codes to define exposure groups using the reason for the termination of a spell (ENDCODE) using the NHSTT dataset 3](#_Toc214978790)

[**Supplementary Table 3.** List of variables used in the analysis. 4](#_Toc214978791)

[**Supplementary Table 4.** Follow-up times (in quarters) for the exposed and non-exposed groups. 6](#_Toc214978792)

[**Supplementary Table 5.** Summary statistics on monthly earnings and employment with and without Inverse Probability Weights (IPWs). 7](#_Toc214978793)

[**Supplementary Figure 1.** Average monthly pay and average monthly pay with IPWs broken down by exposure group 9](#_Toc214978794)

[**Supplementary Figure 2**. Covariate balance plot showing means of variables in the Inverse Probability Weighting (IPW) model, both before and after adjustment 10](#_Toc214978795)

[**Supplementary Table 6.** Effects of NHS TT treatment completion on monthly earnings (£) broken-down by socio-demographic and mental health related characteristics. 11](#_Toc214978796)

[**Supplementary Table 7.** Effects of NHS TT treatment completion on probability of being a paid employee broken-down by socio-demographic and mental health related characteristics. 15](#_Toc214978797)

[**Supplementary Appendix A:** Heterogenous treatment effects 19](#_Toc214978798)

[**Supplementary Appendix B:** Sensitivity analysis: 24](#_Toc214978799)

# **Supplementary Table 1.** Data sources and time periods covered

| **Dataset name** | **Period covered** |
| --- | --- |
| HM Revenue and Customs (HMRC) Pay As You Earn Real Time Information (HMRC PAYE) | 01 April 2014 to 31 December 2022 |
| NHS Talking Therapies (NHSTT) | 01 April 2016 to 31 March 2020 |
| 2011 Census for England and Wales | 2011 |
| ONS death registrations | 2009 to 31 December 2022 |

# **Supplementary Table 2.** Codes to define exposure groups using the reason for the termination of a spell (ENDCODE) using the NHSTT dataset

| **Reason for the termination of the spell definition** | **Codes** |
| --- | --- |
| Suitable for NHSTT treatment, but patient declined treatment that was offered | 14 |
| Completed scheduled treatment | 42 |
| Dropped out of treatment (unscheduled discontinuation) | 43 |

Notes:

1. The reason for the termination as reported by the therapist

# **Supplementary Table 3.** List of variables used in the analysis.

| **Variable name** | **Description** | **Data source** |
| --- | --- | --- |
| **Outcomes** | | |
| Employee status (binary) | Being in paid employment is defined as receiving monthly employee pay greater then £0. | HMRC PAYE |
| Monthly earnings (numerical) | Aggregated quarterly pay as recorded in HMRC Pay As You Earn (PAYE). Records with negative pay are imputed as zero. Pay greater than the 99.9th centile is set to the value at the 99.9th centile. | HMRC PAYE |
| **Other variables** | | |
| Age at referral (numerical) | Age in years on the date of the referral to NHS Talking Therapies, calculated using the date of birth recorded in the 2011 Census. | Census 2011 |
| Age band (categorical) | Age in years on the date of the referral to NHS Talking Therapies, calculated using the date of birth recorded in the 2011 Census and grouped into 4-year age groups. | Census 2011 |
| Caseness (binary) | Flag derived from NHS Talking Therapies to indicate whether the referral was at clinical caseness at first assessment. | NHS Talking Therapies |
| Country of birth (categorical) | Aggregated country of birth derived from country of birth recorded in the 2011 Census. | Census 2011 |
| Dependent children (categorical) | Number of dependent children in the household recorded in the 2011 Census. | Census 2011 |
| Deprivation (categorical) | Dimensions of household deprivation from the 2011 Census based on four selected household characteristics. Employment; Where any member of a household, who is not a full-time student, is either unemployed or long-term sick, Education: No person in the household has at least Level 2 education (see highest level of qualification), and no person aged 16 to 18 is a full-time student. Health and disability: Any person in the household has general health that is 'bad' or 'very bad' or has a long-term health problem, Housing: The household's accommodation is either overcrowded, with an occupancy rating -1 or less, or is in a shared dwelling, or has no central heating. | Census 2011 |
| Deterioration (categorical) | Flag derived from NHS Talking Therapies to indicate that person's symptoms have deteriorated as compared to symptoms at the beginning of therapy. | NHS Talking Therapies |
| Diagnosis (categorical) | Primary problem descriptor grouped using ICD-10 diagnosis codes. Depression (F32 and F33), Agoraphobia (F400), Social phobias (F401), Specific (isolated) phobias (F402), panic disorder [episodic paroxysmal anxiety] (F410), Generalised Anxiety Disorder (F411), Mixed anxiety and depressive disorder (F412), Obsessive-compulsive disorder (F42), Post-traumatic stress disorder (F431), Other anxiety or stress related disorder (Other F40-F43). | NHS Talking Therapies |
| Disability (categorical) | Disability derived from long-term health problem or disability from the 2011 Census. The question from 2011 Census form: “Are your day-to-day activities limited because of a health problem or disability which has lasted, or is expected to last, at least 12 months?” | Census 2011 |
| ENDCODE (categorical) | A classification which identifies the reason for the termination of NHS Talking Therapies spell as determined by the care professional. | NHS Talking Therapies |
| Ethnicity (categorical) | Grouped ethnicity derived from ethnicity recorded in the 2011 Census. | Census 2011 |
| First therapy date (date) | Date of first therapy received by the patient. This is often the second contact appointment with the service as first contact appointment is the assessment. We exclude the assessment and take the actual first therapy date. | NHS Talking Therapies |
| Highest qualification (categorical) | Highest qualification received recorded in the 2011 Census.   - Apprenticeship - Level 1: one to four GCSEs (any grade) or equivalent - Level 2: five or more GCSEs (grades A* to C) or equivalent - Level 3: two or more A levels or equivalent - Level 4 and above: University degrees, Higher National Certificates (HNCs), Higher National Diplomas (HNDs) and professional qualifications like teaching, nursing or accountancy - No qualifications - Other | Census 2011 |
| Medication (categorical) | An indication of whether the patient is taking Psychotropic Medication, as stated by the patient. | NHS Talking Therapies |
| No change (categorical) | Flag derived from NHS Talking Therapies to indicate that person's symptoms have not changed after therapy as compared to symptoms at the beginning of therapy. | NHS Talking Therapies |
| NS-SEC (categorical) | [The National Statistics Socio-economic classification (NS-SEC) derived from the 2011 Census.](https://www.ons.gov.uk/methodology/classificationsandstandards/otherclassifications/thenationalstatisticssocioeconomicclassificationnssecrebasedonsoc2010) | Census 2011 |
| Number of treatment session (numerical) | Number of treatment session received by the patient. | NHS Talking Therapies |
| Region (categorical) | English region of residence. | Census 2011 |
| Reliable improvement (categorical) | Flag derived from NHS Talking Therapies to indicate that person's symptoms have improved by a statistically reliable amount, but the amount falls short of full recovery | NHS Talking Therapies |
| Reliable recovery (categorical) | Flag derived from NHS Talking Therapies to indicate that person's symptoms have improved by a significantly large margin from above a clinical threshold at the start of treatment, to below this threshold at the end of treatment. | NHS Talking Therapies |
| Self-reported employment status (categorical) | Employment status derived from NHS Talking Therapies. Information provided by a patient at the beginning of therapy. | NHS Talking Therapies |
| Sex (categorical) | Sex recorded in the 2011 Census. | Census 2011 |
| Source of referral (categorical) | A classification which identifies the source of referral derived from NHS Talking Therapies. | NHS Talking Therapies |
| Therapy intensity (categorical) | Intensity of the therapy received by the patient. High intensity therapy helps patients with more complex problems related to anxiety and depression. Low intensity therapy equips patients with skills to help them with mild to moderate problems and emphasises self-management of symptoms using self-help books, websites or group sessions. | NHS Talking Therapies |
| Time since first therapy (numerical) | Time period since the quarter in which the first therapy occurred. The time periods before and after first therapy are grouped in four quarterly periods. The four quarters before the first therapy (including the quarter in which the therapy occurred) is the time period to which the outcomes in all other time periods are compared. | NHS Talking Therapies |
| Recovery type (categorical) | Recovery types were predefined by NHSTT and are based on scores from Patient Health Questionnaire (PHQ-9) and Generalised Anxiety Disorder (GAD-7) (or an Anxiety Disorder Specific Measure (ADSM)) taken at the beginning and end of therapy. Grouped recovery types derived from recovery, improvement, deterioration and no change flags to indicate whether the referral has reliably recovered, improved, deteriorated or whether there was no change. | NHS Talking Therapies |

# **Supplementary Table 4.** Follow-up times (in quarters) for the exposed and non-exposed groups.

| **Exposure group** | **Minimum follow-up pre-treatment (in calendar quarters)** | **Maximum follow-up pre-treatment (in calendar quarters)** | **Average follow-up pre-treatment (in calendar quarters)** | **Minimum follow-up post-treatment (in calendar quarters)** | **Maximum follow-up post-treatment (in calendar quarters)** | **Average follow-up post-treatment (in calendar quarters)** |
| --- | --- | --- | --- | --- | --- | --- |
| Completed treatment (exposed group) | -8 | -16 | -13.7 | 1 | 26 | 18.7 |
| Dropped out of treatment (non-exposed group) | -8 | -16 | -13.7 | 1 | 26 | 18.6 |

# **Supplementary Table 5.** Summary statistics on monthly earnings and employment with and without Inverse Probability Weights (IPWs).

| **Time to first therapy (in quarters)** | **Exposed group (completed treatment)** | | | | |  | **Non-exposed group (dropped out of treatment)** | | | | **All** | | | | |
| --- | --- | --- | --- | --- | --- | --- | --- | --- | --- | --- | --- | --- | --- | --- | --- |
|  | Count of people | Average monthly earnings (£) | Average monthly earnings (individuals in employment only) (£) | Average monthly earnings with IPWs (£) | Percentage of individuals in paid employment (%) | Count of people | Average monthly earnings (£) | Average monthly earnings (individuals in employment only) (£) | Average monthly earnings with IPWs (£) | Percentage of individuals in paid employment (%) | Count of people | Proportion of the overall sample (%) | Average monthly earnings (£) | Average monthly earnings (individuals in employment only) (£) | Percentage of individuals in paid employment (%) |
| -16 | 267,914 | 1,732.9 | 2,325.8 | 1,650.1 | 74.5 | 114,327 | 1,368.0 | 2,041.0 | 1,568.2 | 67.0 | 382,241 | 45.4 | 1,623.8 | 2,246.8 | 72.3 |
| -15 | 312,135 | 1,747.9 | 2,344.9 | 1,665.4 | 74.5 | 132,081 | 1,387.8 | 2,068.0 | 1,590.3 | 67.1 | 444,216 | 52.7 | 1,640.8 | 2,268.5 | 72.3 |
| -14 | 353,689 | 1,762.2 | 2,365.8 | 1,679.7 | 74.5 | 149,247 | 1,401.7 | 2,088.4 | 1,605.1 | 67.1 | 502,936 | 59.7 | 1,655.2 | 2,289.4 | 72.3 |
| -13 | 397,040 | 1,770.1 | 2,379.0 | 1,688.0 | 74.4 | 167,496 | 1,413.6 | 2,106.7 | 1,618.5 | 67.1 | 564,536 | 67.0 | 1,664.3 | 2,304.0 | 72.2 |
| -12 | 441,669 | 1,777.5 | 2,394.0 | 1,695.2 | 74.2 | 185,793 | 1,419.8 | 2,119.4 | 1,624.3 | 67.0 | 627,462 | 74.5 | 1,671.6 | 2,318.5 | 72.1 |
| -11 | 488,870 | 1,783.9 | 2,406.9 | 1,702.1 | 74.1 | 204,909 | 1,426.3 | 2,133.7 | 1,629.7 | 66.8 | 693,779 | 82.4 | 1,678.3 | 2,332.0 | 72.0 |
| -10 | 531,687 | 1,790.3 | 2,420.8 | 1,708.4 | 74.0 | 222,685 | 1,433.9 | 2,147.7 | 1,638.0 | 66.8 | 754,372 | 89.6 | 1,685.1 | 2,345.9 | 71.8 |
| -9 | 573,016 | 1,793.6 | 2,429.7 | 1,711.8 | 73.8 | 240,092 | 1,439.5 | 2,159.1 | 1,644.5 | 66.7 | 813,108 | 96.6 | 1,689.1 | 2,355.4 | 71.7 |
| -8 | 593,300 | 1,800.3 | 2,441.5 | 1,718.3 | 73.7 | 248,827 | 1,447.6 | 2,172.8 | 1,651.9 | 66.6 | 842,127 | 100.0 | 1,696.1 | 2,367.7 | 71.6 |
| -7 | 593,300 | 1,806.8 | 2,452.8 | 1,725.3 | 73.7 | 248,827 | 1,456.2 | 2,185.7 | 1,660.6 | 66.6 | 842,127 | 100.0 | 1,703.2 | 2,379.3 | 71.6 |
| -6 | 593,300 | 1,812.6 | 2,465.1 | 1,730.9 | 73.5 | 248,827 | 1,462.4 | 2,198.1 | 1,667.0 | 66.5 | 842,127 | 100.0 | 1,709.1 | 2,391.6 | 71.5 |
| -5 | 593,300 | 1,814.4 | 2,473.1 | 1,732.7 | 73.4 | 248,827 | 1,465.3 | 2,208.1 | 1,669.7 | 66.4 | 842,127 | 100.0 | 1,711.2 | 2,400.2 | 71.3 |
| -4 | 593,300 | 1,815.7 | 2,482.3 | 1,734.0 | 73.1 | 248,827 | 1,467.3 | 2,217.3 | 1,671.9 | 66.2 | 842,127 | 100.0 | 1,712.8 | 2,409.4 | 71.1 |
| -3 | 593,300 | 1,813.0 | 2,487.2 | 1,731.3 | 72.9 | 248,827 | 1,465.5 | 2,227.8 | 1,669.7 | 65.8 | 842,127 | 100.0 | 1,710.3 | 2,416.0 | 70.8 |
| -2 | 593,300 | 1,801.6 | 2,486.5 | 1,719.9 | 72.5 | 248,827 | 1,452.9 | 2,226.9 | 1,657.8 | 65.2 | 842,127 | 100.0 | 1,698.6 | 2,415.3 | 70.3 |
| -1 | 593,300 | 1,766.7 | 2,461.4 | 1,684.9 | 71.8 | 248,827 | 1,418.5 | 2,206.3 | 1,624.0 | 64.3 | 842,127 | 100.0 | 1,663.8 | 2,391.8 | 69.6 |
| **0** | **593,300** | **1,716.6** | **2,421.6** | **1,635.7** | **70.9** | **248,827** | **1,372.9** | **2,165.6** | **1,576.6** | **63.4** | **842,127** | **100.0** | **1,615.0** | **2,351.8** | **68.7** |
| 1 | 593,247 | 1,704.4 | 2,425.0 | 1,627.0 | 70.3 | 248,746 | 1,373.1 | 2,182.9 | 1,568.6 | 62.9 | 841,993 | 100.0 | 1,606.5 | 2,359.0 | 68.1 |
| 2 | 593,121 | 1,701.7 | 2,437.6 | 1,627.4 | 69.8 | 248,611 | 1,376.0 | 2,205.5 | 1,564.1 | 62.4 | 841,732 | 100.0 | 1,605.5 | 2,374.4 | 67.6 |
| 3 | 592,950 | 1,701.8 | 2,451.6 | 1,629.3 | 69.4 | 248,472 | 1,377.1 | 2,221.4 | 1,559.9 | 62.0 | 841,422 | 99.9 | 1,605.9 | 2,388.9 | 67.2 |
| 4 | 592,723 | 1,702.3 | 2,462.3 | 1,631.4 | 69.1 | 248,315 | 1,379.8 | 2,234.5 | 1,559.5 | 61.7 | 841,038 | 99.9 | 1,607.1 | 2,400.3 | 67.0 |
| 5 | 592,480 | 1,707.6 | 2,478.9 | 1,637.6 | 68.9 | 248,151 | 1,384.7 | 2,253.3 | 1,561.6 | 61.5 | 840,631 | 99.8 | 1,612.3 | 2,417.5 | 66.7 |
| 6 | 592,246 | 1,710.9 | 2,491.1 | 1,641.8 | 68.7 | 247,975 | 1,389.7 | 2,269.1 | 1,564.3 | 61.2 | 840,221 | 99.8 | 1,616.1 | 2,430.7 | 66.5 |
| 7 | 591,995 | 1,713.3 | 2,503.5 | 1,645.2 | 68.4 | 247,815 | 1,394.5 | 2,283.3 | 1,567.4 | 61.1 | 839,810 | 99.7 | 1,619.2 | 2,443.6 | 66.3 |
| 8 | 591,723 | 1,716.2 | 2,513.1 | 1,648.8 | 68.3 | 247,653 | 1,401.4 | 2,300.6 | 1,572.2 | 60.9 | 839,376 | 99.7 | 1,623.3 | 2,455.4 | 66.1 |
| 9 | 591,430 | 1,718.0 | 2,521.8 | 1,651.4 | 68.1 | 247,472 | 1,407.1 | 2,315.1 | 1,576.1 | 60.8 | 838,902 | 99.6 | 1,626.3 | 2,465.6 | 66.0 |
| 10 | 590,913 | 1,721.7 | 2,535.1 | 1,656.0 | 67.9 | 247,097 | 1,413.0 | 2,330.7 | 1,579.5 | 60.6 | 838,010 | 99.5 | 1,630.7 | 2,479.6 | 65.8 |
| 11 | 583,394 | 1,724.6 | 2,545.7 | 1,659.8 | 67.7 | 243,414 | 1,420.2 | 2,345.1 | 1,584.9 | 60.6 | 826,808 | 98.2 | 1,635.0 | 2,491.2 | 65.6 |
| 12 | 570,945 | 1,727.9 | 2,557.0 | 1,664.0 | 67.6 | 236,942 | 1,426.1 | 2,357.3 | 1,588.9 | 60.5 | 807,887 | 95.9 | 1,639.4 | 2,502.9 | 65.5 |
| 13 | 556,816 | 1,730.4 | 2,567.7 | 1,667.5 | 67.4 | 229,638 | 1,434.3 | 2,372.1 | 1,596.3 | 60.5 | 786,454 | 93.4 | 1,644.0 | 2,514.9 | 65.4 |
| 14 | 532,697 | 1,730.2 | 2,576.4 | 1,668.0 | 67.2 | 218,050 | 1,436.6 | 2,382.8 | 1,596.4 | 60.3 | 750,747 | 89.1 | 1,644.9 | 2,524.4 | 65.2 |
| 15 | 492,083 | 1,719.3 | 2,577.3 | 1,657.8 | 66.7 | 200,902 | 1,432.1 | 2,391.0 | 1,590.6 | 59.9 | 692,985 | 82.3 | 1,636.0 | 2,527.3 | 64.7 |
| 16 | 447,697 | 1,708.8 | 2,580.2 | 1,647.8 | 66.2 | 183,294 | 1,427.5 | 2,394.4 | 1,584.3 | 59.6 | 630,991 | 74.9 | 1,627.1 | 2,530.2 | 64.3 |
| 17 | 405,556 | 1,703.6 | 2,582.5 | 1,643.0 | 66.0 | 166,877 | 1,426.2 | 2,400.2 | 1,582.1 | 59.4 | 572,433 | 68.0 | 1,622.7 | 2,533.2 | 64.1 |
| 18 | 362,555 | 1,697.9 | 2,583.9 | 1,637.3 | 65.7 | 149,357 | 1,423.7 | 2,406.8 | 1,579.7 | 59.2 | 511,912 | 60.8 | 1,617.9 | 2,536.0 | 63.8 |
| 19 | 318,584 | 1,693.7 | 2,582.1 | 1,632.9 | 65.6 | 131,544 | 1,420.4 | 2,398.9 | 1,575.6 | 59.2 | 450,128 | 53.5 | 1,613.8 | 2,532.3 | 63.7 |
| 20 | 274,064 | 1,689.7 | 2,581.4 | 1,629.2 | 65.5 | 113,750 | 1,417.3 | 2,395.5 | 1,571.0 | 59.2 | 387,814 | 46.1 | 1,609.8 | 2,530.7 | 63.6 |
| 21 | 232,488 | 1,681.8 | 2,579.3 | 1,622.2 | 65.2 | 96,675 | 1,418.5 | 2,400.9 | 1,573.1 | 59.1 | 329,163 | 39.1 | 1,604.4 | 2,530.5 | 63.4 |
| 22 | 189,464 | 1,673.6 | 2,575.7 | 1,614.4 | 65.0 | 78,682 | 1,410.6 | 2,394.1 | 1,563.2 | 58.9 | 268,146 | 31.8 | 1,596.4 | 2,526.0 | 63.2 |
| 23 | 145,636 | 1,670.8 | 2,572.3 | 1,612.4 | 65.0 | 60,707 | 1,408.1 | 2,394.8 | 1,559.9 | 58.8 | 206,343 | 24.5 | 1,593.5 | 2,523.7 | 63.1 |
| 24 | 99,743 | 1,669.7 | 2,573.9 | 1,610.8 | 64.9 | 42,115 | 1,416.3 | 2,414.6 | 1,570.3 | 58.7 | 141,858 | 16.8 | 1,594.4 | 2,529.9 | 63.0 |
| 25 | 58,513 | 1,665.5 | 2,580.5 | 1,606.3 | 64.5 | 24,968 | 1,414.3 | 2,422.2 | 1,569.5 | 58.4 | 83,481 | 9.9 | 1,590.3 | 2,536.4 | 62.7 |
| 26 | 19,192 | 1,666.8 | 2,575.3 | 1,606.8 | 64.7 | 8,348 | 1,435.1 | 2,444.9 | 1,575.8 | 58.7 | 27,540 | 3.3 | 1,596.6 | 2,538.4 | 62.9 |

Notes:

1. To calculate Average monthly earnings for individuals in employment only the dataset was restricted to include only the months for which individuals were a paid employee

# **Supplementary Figure 1.** Average monthly pay and average monthly pay with IPWs broken down by exposure group


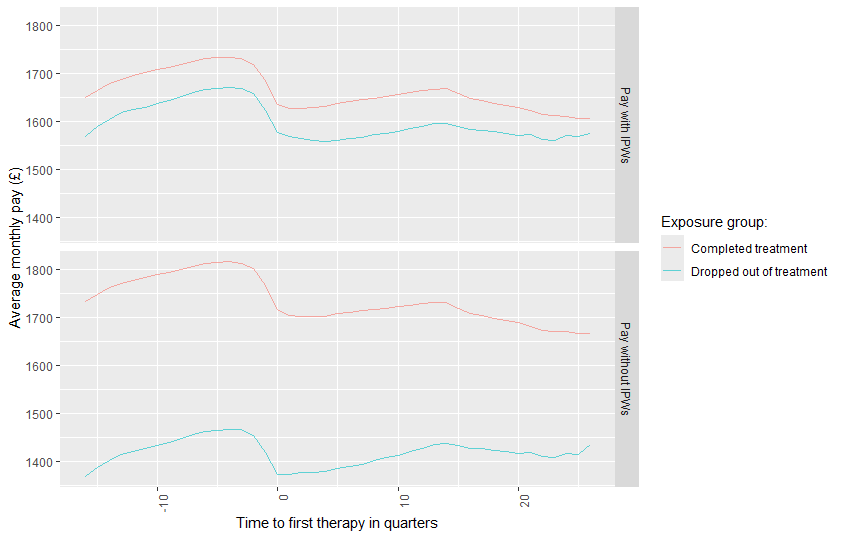


# **Supplementary Figure 2**. Covariate balance plot showing means of variables in the Inverse Probability Weighting (IPW) model, both before and after adjustment


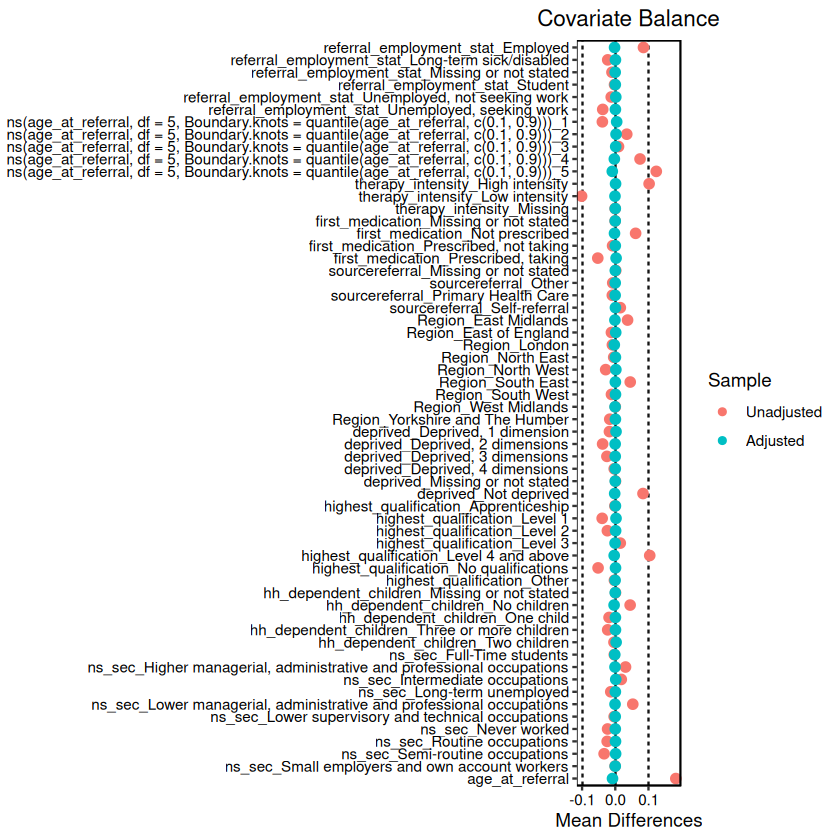


# **Supplementary Table 6.** Effects of NHS TT treatment completion on monthly earnings (£) broken-down by socio-demographic and mental health related characteristics.

| **Characteristic** | **Category** | **Time since first therapy in years** | | | | | | | | | | |
| --- | --- | --- | --- | --- | --- | --- | --- | --- | --- | --- | --- | --- |
|  |  | **-4** | **-3** | **-2** | **-1** | **1** | **2** | **3** | **4** | **5** | **6** | **7** |
| **Age band** | 25-34 | -24.5**  (-37.7--11.4) | -14.0**  (-23.8--4.2) | 0.4  (-6.9-7.8) | 2.6  (-2.7-7.9) | 14.7**  (8.9-20.5) | 31.6**  (23.7-39.4) | 31.7**  (22.6-40.8) | 30.2**  (19.8-40.5) | 24.8**  (12.2-37.4) | 18.1*  (1.6-34.6) | 13.6  (-16.9-44.0) |
|  | 35-44 | 24.8**  (8.0-41.6) | 12.9*  (0.6-25.3) | 6.4  (-2.8-15.7) | 5.2  (-1.5-11.9) | 8.2 *  (0.6-15.8) | 25.4**  (15.4-35.4) | 32.3**  (21.1-43.6) | 35.9**  (23.2-48.6) | 41.3**  (26.4-56.2) | 45.5**  (26.7-64.4) | 44.6**  (10.9-78.3) |
|  | 45-54 | 15.8  (-1.5-33.2) | 15.0*  (2.2-27.8) | 10.1*  (0.8-19.5) | -0.9  (-7.6-5.8) | -3.3  (-11.6-5.0) | 0.8  (-10.4-11.9) | -4.5  (-17.1-8.0) | -13.8  (-27.8-0.1) | -16.8*  (-33.2-0.5) | -22.2*  (-43.0--1.4) | -25.5  (-62.0-11.0) |
|  | 55-60 | 24.0  (-0.9-48.9) | 8.1  (-10.8-27.0) | 12.1  (-1.2-25.3) | 5.5  (-3.9-14.8) | -20.9**  (-33.6--8.2) | -25.7**  (-43.6--7.8) | -35.8**  (-56.1--15.6) | -40.8**  (-63.8--17.9) | -42.7**  (-69.7--15.6) | -57.7**  (-94.6--20.7) | -37.4  (-107.6-32.8) |
| **Deprivation** | 1 dimension | -3.1  (-17.8-11.6) | 3.6  (-7.3-14.5) | 7.2  (-0.8-15.3) | 4.1  (-1.6-9.9) | 6.9*  (0.1-13.7) | 19.8**  (10.6-29.0) | 18.5**  (8.0-29.0) | 17.1**  (5.3-29.0) | 16.0*  (1.9-30.1) | 23.7**  (5.7-41.6) | 36.5*  (3.8-69.1) |
|  | 2 dimensions | 2.7  (-13.2-18.7) | 4.2  (-7.5-16.0) | 2.3  (-6.2-10.9) | 2.9  (-3.2-9.0) | -2.4  (-9.4-4.5) | 2.7  (-6.7-12.1) | 6.3  (-4.4-17.1) | 5.0  (-7.2-17.1) | 1.4  (-13.0-15.8) | 0.4  (-18.2-19.0) | 3.2  (-29.4-35.7) |
|  | 3 dimensions | 0.6  (-20.1-21.3) | 3.8  (-10.6-18.2) | 9.5  (-0.9-19.8) | 0.4  (-7.0-7.9) | 0.6  (-7.6-8.9) | 10.2  (-1.3-21.7) | 13.9*  (0.9-26.8) | 15.2*  (0.6-29.8) | 17.3*  (0.5-34.2) | 16.7  (-4.8-38.1) | 34.6  (-4.0-73.2) |
|  | 4 dimensions | 31.7  (-15.9-79.3) | 1.5  (-35.0-38.0) | 7.7  (-17.5-32.9) | 2.8  (-14.2-19.9) | 4.0  (-16.9-24.9) | 10.4  (-18.4-39.2) | 22.4  (-11.4-56.2) | 30.9  (-6.5-68.3) | 30.4  (-13.1-73.9) | 50.7  (-4.0-105.3) | 60.5  (-28.9-149.9) |
|  | Not deprived | 7.0  (-6.5-20.4) | 2.2  (-7.7-12.2) | 5.3  (-2.1-12.7) | 1.5  (-3.8-6.8) | 5.7  (-0.6-12.0) | 18.4**  (9.8-26.9) | 17.2**  (7.6-26.8) | 13.6*  (2.7-24.4) | 13.5*  (0.7-26.4) | 3.0  (-13.6-19.7) | -7.4  (-37.3-22.5) |
| **Diagnosis** | Agoraphobia | -42.1  (-119.9-35.8) | 21.0  (-29.2-71.2) | 11.6  (-26.4-49.7) | -0.6  (-28.9-27.6) | -11.0  (-42.3-20.2) | -17.0  (-59.6-25.6) | -12.0  (-60.8-36.8) | -25.9  (-80.5-28.6) | -15.2  (-80.6-50.1) | 6.3  (-79.3-91.8) | -53.5  (-216.7-109.8) |
|  | Depression | 3.6  (-9.5-16.8) | 5.0  (-5.0-15.0) | 8.1*  (0.5-15.7) | 3.5  (-1.9-9.0) | 7.0*  (0.6-13.4) | 23.4**  (14.8-32.0) | 23.3**  (13.5-33.2) | 19.3**  (8.2-30.4) | 22.3**  (8.9-35.6) | 21.4*  (3.7-39.1) | 22.0  (-12.1-56.0) |
|  | GAD | 11.7  (-5.2-28.7) | 5.6  (-7.0-18.1) | 8.5  (-1.0-18.0) | 2.3  (-4.7-9.3) | 3.6  (-4.8-12.1) | 12.7*  (1.5-23.9) | 12.7  (0.0-25.4) | 13.4  (-1.2-28.0) | 6.8  (-10.9-24.6) | 5.9  (-18.2-30.0) | -4.8  (-50.5-40.8) |
|  | Mixed anxiety and depressive disorder | 11.6  (-23.3-46.6) | -1.8  (-24.6-21.0) | 4.6  (-10.1-19.4) | -0.6  (-11.3-10.0) | 1.3  (-11.4-14.0) | 4.7  (-12.4-21.9) | 8.8  (-10.4-27.9) | 14.2  (-7.1-35.4) | 10.3  (-13.4-34.0) | 3.7  (-24.4-31.9) | 13.1  (-30.5-56.8) |
|  | OCD | 28.9  (-26.9-84.6) | 28.5  (-13.8-70.8) | 27.1  (-3.6-57.7) | 24.9*  (2.5-47.2) | 12.0  (-12.4-36.3) | 4.6  (-29.4-38.6) | -2.1  (-40.7-36.5) | 0.3  (-44.0-44.6) | -22.7  (-75.6-30.1) | 4.7  (-65.0-74.4) | -44.4  (-185.0-96.1) |
|  | Other anxiety or stress related disorder | 8.4  (-33.9-50.6) | 8.6  (-23.9-41.1) | -1.0  (-25.3-23.3) | 6.5  (-10.8-23.9) | 20.4  (-1.2-42.0) | 21.8  (-7.7-51.2) | 31.8  (-1.2-64.8) | 18.4  (-17.7-54.6) | 16.3  (-28.1-60.7) | 16.8  (-42.4-75.9) | 7.4  (-109.2-123.9) |
|  | Panic disorder | -31.4  (-84.2-21.4) | -23.0  (-59.3-13.2) | -14.9  (-41.4-11.7) | -7.8  (-26.6-11.0) | 27.1**  (6.5-47.7) | 46.6**  (16.8-76.5) | 34.9*  (0.1-69.7) | 38.6*  (0.2-76.9) | 71.4**  (26.1-116.6) | 82.5**  (25.6-139.4) | 49.2  (-56.7-155.1) |
|  | PTSD | 7.3  (-32.4-47.0) | -1.6  (-31.3-28.0) | 11.5  (-11.0-34.0) | 5.4  (-10.8-21.6) | -9.2  (-26.5-8.2) | 3.3  (-21.5-28.0) | -12.1  (-40.4-16.2) | -5.3  (-38.5-27.9) | -16.2  (-56.4-24.0) | -58.9*  (-111.4--6.4) | -82.4  (-193.5-28.7) |
|  | Social phobias | -5.1  (-63.2-53.0) | -19.9  (-61.4-21.5) | 3.3  (-27.4-34.1) | 10.7  (-12.6-34.0) | -3.7  (-27.7-20.3) | -0.3  (-34.6-34.0) | 6.2  (-32.3-44.6) | 30.2  (-13.4-73.8) | 42.3  (-10.5-95.1) | 8.5  (-59.2-76.2) | -33.2  (-167.6-101.3) |
|  | Specific (isolated) phobias | 21.6  (-91.9-135.0) | 30.9  (-41.8-103.5) | 6.3  (-44.3-56.9) | -1.0  (-38.8-36.9) | 25.1  (-14.1-64.3) | 30.8  (-28.7-90.2) | 20.3  (-48.3-89.0) | -21.1  (-99.2-57.0) | -41.5  (-136.2-53.1) | -61.1  (-191.2-69.0) | -148.2  (-377.2-80.9) |
|  | Missing or not stated | -20.2  (-46.5-6.1) | -3.2  (-22.5-16.1) | -0.6  (-13.9-12.6) | 2.2  (-7.3-11.6) | -1.9  (-12.6-8.8) | 14.1  (-0.6-28.8) | 14.5  (-2.0-31.0) | 5.4  (-13.2-24.1) | 5.1  (-16.3-26.6) | -0.6  (-26.7-25.5) | 18.4  (-24.1-60.9) |
| **Employment status** | Employed | 1.2  (-9.7-12.1) | 0.1  (-7.9-8.1) | 6.1*  (0.2-11.9) | 2.9  (-1.3-7.2) | 10.2**  (4.9-15.4) | 19.6**  (12.4-26.8) | 17.2**  (9.0-25.3) | 11.8*  (2.6-21.0) | 10.1  (-0.8-21.1) | 5.4  (-8.8-19.6) | -0.1  (-25.4-25.2) |
|  | Not working, long-term sick/disabled | 20.6  (1.2-40.0) | 12.1*  (-2.7-26.9) | 3.5*  (-7.6-14.6) | 1.0  (-6.7-8.7) | -15.9**  (-22.9--8.9) | -12.5**  (-21.6--3.4) | -11.1*  (-21.2--1.0) | -8.4  (-19.8-3.1) | -4.0  (-17.3-9.3) | -15.7  (-32.9-1.5) | -23.2  (-52.7-6.4) |
|  | Student | -11.9  (-78.8-55.1) | 24.5  (-29.2-78.2) | 8.0  (-35.4-51.5) | -1.7  (-36.4-33.0) | 1.4  (-31.9-34.6) | 28.0  (-22.0-78.0) | 84.3**  (26.4-142.1) | 92.4**  (26.9-157.8) | 74.2  (-3.9-152.3) | 107.3*  (9.8-204.8) | 124.5  (-75.6-324.7) |
|  | Not working, not seeking work | 53.8**  (29.6-78.0) | 25.1*  (5.8-44.5) | 16.0*  (1.8-30.2) | 2.1  (-8.2-12.4) | -11.9*  (-21.6--2.1) | 6.4  (-5.3-18.2) | 11.1  (-2.0-24.2) | 15.9*  (1.1-30.8) | 9.8  (-8.4-28.0) | 17.2  (-5.9-40.3) | -6.4  (-53.8-40.9) |
|  | Not working, seeking work | 17.3  (-9.6-44.2) | 25.3*  (4.6-46.1) | 26.1**  (9.6-42.6) | 17.7**  (4.6-30.7) | -5.6  (-18.8-7.6) | 21.5**  (6.4-36.6) | 25.6**  (9.2-42.0) | 35.2**  (17.2-53.2) | 45.7**  (25.2-66.2) | 45.4**  (19.2-71.6) | 63.0**  (17.7-108.3) |
|  | Missing or not stated | -24.8*  (-49.5--0.1) | -18.7  (-37.6-0.2) | -17.3*  (-30.9--3.6) | -10.7*  (-20.6--0.9) | 4.9  (-6.3-16.0) | 17.2*  (2.2-32.1) | 15.7  (-1.4-32.9) | 13.1  (-6.1-32.3) | 6.5  (-16.5-29.5) | -6.7  (-35.5-22.2) | 27.6  (-26.6-81.7) |
| **Ethnicity** | Asian | -37.7  (-80.0-4.7) | -19.4  (-52.0-13.2) | 8.3  (-15.8-32.3) | -3.2  (-20.4-14.1) | 21.2*  (0.2-42.1) | 38.9**  (11.3-66.4) | 39.4*  (8.5-70.4) | 26.4  (-7.7-60.4) | 21.3  (-18.9-61.6) | 14.1  (-38.1-66.3) | 87.3  (-12.9-187.5) |
|  | Black | 31.3  (-20.6-83.3) | 21.9  (-18.1-61.9) | 7.1  (-23.9-38.1) | 4.5  (-17.7-26.8) | -4.2  (-31.1-22.8) | -15.0  (-51.5-21.4) | -10.9  (-50.8-29.0) | -25.8  (-71.3-19.7) | -38.2  (-92.3-15.9) | -38.9  (-106.2-28.5) | -86.8  (-211.5-37.9) |
|  | Mixed | -48.8  (-109.6-12.0) | -8.8  (-53.7-36.2) | 10.8  (-21.9-43.5) | 5.1  (-18.2-28.5) | -1.8  (-30.6-27.1) | 15.9  (-22.8-54.7) | 37.7  (-5.3-80.6) | 19.3  (-27.8-66.4) | -9.3  (-66.4-47.9) | 41.5  (-35.1-118.1) | 111.8  (-18.5-242.1) |
|  | Other | -58.3  (-165.1-48.5) | -49.4  (-130.5-31.7) | -39.8  (-97.9-18.4) | -19.7  (-62.4-23.1) | -16.9  (-63.8-30.0) | -11.7  (-79.5-56.0) | -49.7  (-128.6-29.2) | 2.6  (-81.4-86.7) | 71.8  (-23.1-166.7) | 34.9  (-92.5-162.2) | -68.2  (-288.8-152.3) |
|  | White | 5.8  (-3.1-14.7) | 3.8  (-2.8-10.3) | 5.8*  (1.0-10.6) | 3.1  (-0.4-6.6) | 4.7*  (0.6-8.8) | 16.9**  (11.4-22.4) | 16.8**  (10.6-23.1) | 16.1**  (9.0-23.2) | 16.3**  (7.8-24.7) | 12.1*  (1.2-22.9) | 7.8  (-11.7-27.2) |
| **Highest qualification** | Apprenticeship | 5.6  (-50.4-61.7) | 4.8  (-37.4-47.0) | -8.5  (-39.6-22.7) | 5.4  (-16.4-27.2) | 7.6  (-20.1-35.3) | -1.5  (-38.7-35.8) | 31.9  (-11.7-75.4) | 40.7  (-9.3-90.7) | 23.4  (-36.2-82.9) | 48.8  (-29.4-127.0) | 40.7  (-109.7-191.2) |
|  | Level 1 | -3.9  (-18.8-11.0) | -1.0  (-12.1-10.0) | 0.7  (-7.5-8.9) | 1.9  (-3.9-7.8) | 4.6  (-2.1-11.2) | 7.2  (-2.0-16.3) | 10.0  (-0.6-20.7) | 2.8  (-9.4-14.9) | 0.5  (-13.8-14.8) | 3.1  (-15.4-21.7) | 5.2  (-26.9-37.3) |
|  | Level 2 | -29.2**  (-44.7--13.7) | -13.0*  (-24.3--1.7) | -1.9 (-10.1-6.3) | -4.0  (-9.9-1.9) | 7.9*  (1.2-14.5) | 22.2**  (13.1-31.4) | 16.3**  (5.8-26.9) | 9.6  (-2.4-21.6) | 6.3 (-8.0-20.6) | -2.7  (-21.2-15.8) | 15.8  (-18.8-50.3) |
|  | Level 3 | 9.9  (-8.5-28.4) | -0.5  (-13.8-12.9) | 3.5 (-6.4-13.5) | 0.8  (-6.4-7.9) | 6.5  (-1.5-14.5) | 19.4**  (8.3-30.5) | 19.2**  (6.5-31.8) | 22.3**  (8.0-36.5) | 26.3**  (9.1-43.6) | 14.0  (-8.4-36.4) | -17.1  (-58.2-24.0) |
|  | Level 4 and above | 35.2**  (14.8-55.7) | 21.7**  (6.5-36.8) | 18.1** (7.1-29.2) | 10.4*  (2.3-18.4) | -1.0  (-10.6-8.6) | 14.5*  (1.7-27.3) | 13.0  (-1.3-27.4) | 9.6  (-6.3-25.6) | 7.3  (-11.4-26.0) | 7.1  (-16.6-30.9) | 12.7  (-29.3-54.8) |
|  | No qualifications | 15.1*  (0.3-29.8) | 11.9*  (1.0-22.9) | 10.8** (2.9-18.8) | 2.7  (-2.9-8.4) | 4.6  (-1.8-10.9) | 5.6  (-3.0-14.2) | 6.3  (-3.7-16.3) | 6.4  (-5.1-17.8) | 5.5  (-8.0-19.0) | -0.8  (-17.9-16.4) | -10.5  (-39.8-18.9) |
|  | Other | -21.1  (-64.5-22.3) | -11.7  (-44.8-21.4) | -3.4  (-27.2-20.4) | -3.3  (-20.6-14.0) | -6.5  (-27.4-14.3) | 2.3  (-25.4-29.9) | 0.5  (-30.8-31.8) | 1.3  (-34.4-37.0) | 7.5  (-34.2-49.2) | 13.2  (-39.6-66.0) | -15.0  (-106.7-76.8) |
| **Medication** | Not prescribed | 7.5  (-6.9-22.0) | 3.3  (-7.4-14.0) | 7.7  (-0.3-15.6) | 4.8  (-1.0-10.5) | 2.6  (-4.0-9.2) | 14.2**  (5.3-23.1) | 16.5**  (6.3-26.6) | 19.3**  (7.8-30.8) | 21.8**  (8.0-35.6) | 19.0*  (1.0-37.0) | 3.0  (-29.0-35.1) |
|  | Prescribed, not taking | -13.1  (-53.0-26.8) | -10.5  (-39.8-18.7) | 3.3  (-18.1-24.7) | 4.1  (-11.3-19.5) | 4.4  (-14.1-22.8) | 25.2*  (0.5-49.8) | 27.0  (-1.2-55.2) | 18.4  (-13.3-50.2) | 20.3  (-16.1-56.8) | 36.8  (-9.7-83.4) | 104.4*  (24.9-184.0) |
|  | Prescribed, taking | 3.9  (-7.1-14.8) | 4.8  (-3.3-12.9) | 5.0  (-1.0-10.9) | 1.5  (-2.8-5.8) | 7.6**  (2.4-12.8) | 18.9**  (11.9-25.9) | 17.3**  (9.4-25.3) | 12.6**  (3.7-21.5) | 10.6*  (0.1-21.1) | 6.5  (-6.9-19.9) | 9.2  (-15.5-33.9) |
|  | Missing or not stated | -34.9*  (-69.7--0.1) | -19.4  (-47.2-8.5) | -5.8  (-26.0-14.4) | -10.4  (-24.7-3.9) | 0.3  (-15.9-16.5) | 10.1  (-12.1-32.2) | 1.9  (-23.4-27.1) | -9.9  (-38.9-19.1) | -27.6  (-61.8-6.7) | -44.7*  (-87.5--1.8) | -29.7  (-104.9-45.5) |
| **NS-SEC** | Full-Time students | -60.0**  (-84.5--35.5) | -38.8**  (-57.9--19.6) | -4.5  (-19.5-10.5) | 0.5  (-10.4-11.4) | 19.6**  (7.8-31.4) | 43.1**  (26.7-59.5) | 45.9**  (26.9-64.9) | 44.2**  (21.8-66.7) | 36.4*  (7.5-65.3) | 27.9  (-12.8-68.5) | 14.1  (-67.2-95.3) |
|  | Higher managerial, administrative and professional | 22.9  (-29.2-75.0) | 17.3  (-20.7-55.4) | 11.6  (-16.5-39.7) | -3.9  (-24.1-16.3) | 14.4  (-10.3-39.1) | 38.5*  (6.0-71.0) | 16.6  (-19.5-52.7) | 6.1  (-33.6-45.7) | 9.6  (-36.8-55.9) | 8.3  (-50.9-67.4) | 15.3  (-90.2-120.7) |
|  | Intermediate occupations | 17.5  (-0.4-35.3) | 11.5  (-1.4-24.5) | 5.0  (-4.5-14.4) | 0.5  (-6.2-7.3) | 8.7*  (0.9-16.6) | 10.3  (-0.2-20.9) | 9.4  (-2.8-21.6) | 8.6  (-5.2-22.5) | 3.3  (-12.9-19.6) | 4.0  (-16.9-25.0) | -4.2  (-41.3-33.0) |
|  | Long-term unemployed | 7.0  (-26.3-40.3) | 3.0  (-21.6-27.6) | 2.4  (-15.4-20.3) | 11.1  (-1.8-23.9) | -1.9  (-15.6-11.7) | -0.4  (-19.2-18.5) | 14.4  (-7.6-36.4) | 19.4  (-7.5-46.2) | 24.0  (-6.3-54.3) | 27.6  (-8.8-64.0) | 30.8  (-33.5-95.0) |
|  | Lower managerial, administrative and professional | 33.9**  (12.5-55.3) | 14.1  (-1.6-29.8) | 11.4  (-0.1-22.9) | 6.4  (-1.9-14.8) | -9.0  (-19.1-1.0) | 10.4  (-3.2-24.0) | 10.6  (-4.6-25.8) | 6.7  (-10.3-23.7) | 9.9  (-9.9-29.8) | -3.8  (-28.9-21.3) | 16.1  (-28.6-60.9) |
|  | Lower supervisory and technical occupations | 22.0  (-8.2-52.3) | 17.7  (-4.8-40.3) | 5.5  (-11.2-22.2) | 6.5  (-5.3-18.4) | 10.8  (-3.4-25.0) | 14.2  (-5.1-33.4) | 11.5  (-10.6-33.5) | 12.1  (-12.9-37.2) | 20.0  (-9.8-49.8) | 8.5  (-29.9-46.9) | -32.6  (-99.8-34.6) |
|  | Never worked | -10.4  (-29.8-9.0) | -6.2  (-20.1-7.8) | -6.9  (-17.1-3.4) | -2.5*  (-9.6-4.7) | 1.2  (-6.6-9.0) | 3.2**  (-7.8-14.2) | 4.0**  (-8.9-17.0) | 7.9*  (-7.3-23.0) | 14.1  (-3.5-31.7) | 14.4  (-8.5-37.3) | 24.8  (-18.8-68.4) |
|  | Routine occupations | 14.1  (-5.1-33.3) | 13.3  (-0.9-27.5) | 9.7  (-0.7-20.1) | 7.4  (0.0-14.8) | 6.4  (-1.6-14.5) | 14.9  (3.9-25.9) | 18.5  (5.7-31.3) | 16.9  (2.4-31.4) | 14.1  (-3.1-31.3) | 7.1  (-15.4-29.5) | -3.2  (-42.6-36.3) |
|  | Semi-routine occupations | 7.0  (-7.5-21.4) | 9.4  (-1.1-19.9) | 9.8*  (2.2-17.4) | 5.1  (-0.3-10.4) | 3.0  (-3.0-9.0) | 13.6**  (5.5-21.8) | 17.9**  (8.4-27.3) | 18.0**  (7.2-28.7) | 17.7**  (5.0-30.5) | 21.8**  (5.6-38.0) | 2.1  (-27.2-31.3) |
|  | Small employers and own account workers | -19.4  (-51.1-12.2) | -19.0  (-41.7-3.6) | -2.7  (-19.3-13.8) | -8.6  (-20.8-3.6) | 1.9  (-11.5-15.3) | -3.2  (-21.1-14.7) | -1.0  (-21.9-19.9) | -5.1  (-28.5-18.2) | -18.3  (-45.8-9.1) | -6.6  (-41.4-28.1) | 14.7  (-46.0-75.4) |
| **Number of treatment sessions** | 2 | 14.9  (-7.8-37.6) | 8.4  (-8.3-25.1) | 8.9  (-3.4-21.1) | 1.3  (-7.5-10.1) | 2.2  (-8.6-13) | 6.4  (-7.7-20.6) | 2.9  (-13.2-19) | 6.2  (-12-24.4) | -5.5  (-26.9-16) | -26.4  (-54.1-1.3) | -41.0  (-90.4-8.5) |
|  | 3 to 5 | 8.7  (-5.4-22.9) | 5.8  (-4.7-16.2) | 6.6  (-1.1-14.3) | 4.1  (-1.4-9.6) | 7.9  (1.2-14.7) | 19.3**  (10.2-28.5) | 17.5**  (7.1-28) | 14.7*  (2.8-26.6) | 13.7  (-0.5-27.9) | 11.3  (-7.3-29.9) | 16.9  (-16.7-50.6) |
|  | 6 to 8 | 12.1  (-9.5-33.6) | 13.3  (-3.0-29.7) | 10.0  (-1.9-21.9) | 2.0  (-6.5-10.4) | 22.5**  (12.0-33.0) | 39.8**  (25.6-54.1) | 40.3**  (24.1-56.4) | 38.8**  (20.5-57.1) | 39.5**  (18.2-60.7) | 33.0*  (6.6-59.4) | 33.3  (-11.9-78.5) |
|  | 9 to 15 | 9.8  (-19.0-38.5) | -1.8  (-23.5-19.8) | 3.1  (-12.9-19.2) | -4.2  (-16.0-7.6) | 26.7**  (14.0-39.3) | 33.4**  (16.1-50.7) | 25.5*  (5.8-45.2) | 24.0*  (2.0-45.9) | 20.3  (-6.5-47.1) | 14.9  (-19.3-49.1) | 20.8  (-43.0-84.5) |
|  | 16 or more | 51.9  (-33.0-136.8) | 1.1  (-52.0-54.2) | 7.2  (-26.8-41.3) | 11.6  (-12.4-35.7) | 30.9*  (1.6-60.2) | 65.1**  (23.3-107.0) | 57.4*  (10.1-104.7) | 46.9  (-4.0-97.7) | 45.2  (-9.8-100.1) | 45.8  (-20.5-112.2) | 16.7  (-90.2-123.6) |
| **Region** | East Midlands | 1.0  (-24.9-26.9) | -2.1  (-21.4-17.2) | 10.8  (-3.5-25.1) | 1.8  (-8.5-12.2) | 5.0  (-8.0-18.0) | 6.1  (-11.4-23.6) | 12.2  (-7.2-31.7) | 8.0  (-14.3-30.3) | 7.3  (-19.5-34.0) | -7.7  (-42.8-27.3) | -29.9  (-97.2-37.3) |
|  | East of England | 28.4*  (1.5-55.3) | 6.3  (-13.9-26.4) | 5.7  (-8.9-20.4) | 4.0  (-6.7-14.7) | -9.2  (-21.1-2.8) | -2.9  (-19.0-13.1) | -5.0  (-23.5-13.5) | 2.8  (-18.1-23.6) | -7.0  (-32.2-18.2) | -7.3  (-39.4-24.8) | 1.8  (-54.6-58.2) |
|  | London | 3.8  (-23.7-31.4) | 11.9  (-9.0-32.7) | 5.7  (-9.9-21.3) | 2.6  (-8.7-13.9) | 9.5  (-3.7-22.7) | 31.3**  (13.7-48.8) | 30.0**  (10.6-49.4) | 29.1**  (7.3-50.8) | 43.3**  (17.5-69.1) | 52.7**  (19.4-86.0) | 62.7*  (3.0-122.5) |
|  | North East | 27.2  (-2.4-56.8) | 4.2  (-16.4-24.8) | 0.6  (-14.5-15.7) | 4.6  (-5.7-15.0) | 8.6  (-3.9-21.0) | 22.4*  (5.2-39.6) | 27.2**  (6.9-47.6) | 18.4  (-4.4-41.3) | 5.7  (-20.3-31.7) | -37.4*  (-70.2--4.6) | -79.2**  (-132.7--25.7) |
|  | North West | 0.4  (-18.2-19.1) | 2.1  (-11.8-16.0) | 6.1  (-4.1-16.3) | 3.2  (-4.2-10.6) | 6.7  (-2.0-15.4) | 14.6*  (2.8-26.5) | 7.1  (-6.6-20.7) | 10.3  (-5.3-25.9) | 17.1  (-1.6-35.7) | 19.2  (-4.6-43.0) | 14.6  (-29.6-58.9) |
|  | South East | 0.2  (-24.4-24.7) | -10.5  (-28.5-7.5) | 0.9  (-12.1-14.0) | -7.6  (-17.2-1.9) | 10.3  (-0.6-21.2) | 28.7**  (13.8-43.6) | 33.7**  (16.7-50.7) | 23.8*  (4.7-42.9) | 22.9*  (0.5-45.4) | 21.2  (-7.4-49.8) | 15.6  (-35.0-66.3) |
|  | South West | -24.1  (-48.5-0.3) | -10.3  (-28.3-7.6) | -11.8  (-25.0-1.4) | 1.7  (-7.7-11.0) | -9.2  (-20.1-1.7) | -6.7  (-21.5-8.1) | -6.9  (-23.9-10.2) | -11.8  (-31.0-7.4) | -16.2  (-39.0-6.6) | 9.2  (-20.9-39.3) | 16.8  (-38.3-72.0) |
|  | West Midlands | 0.5  (-24.3-25.3) | 7.7  (-11.0-26.5) | 14.7*  (0.9-28.5) | 12.5*  (2.7-22.4) | 11.5  (-0.9-23.9) | 27.3**  (10.8-43.9) | 21.2*  (2.2-40.2) | 17.9  (-3.6-39.3) | 5.1  (-20.1-30.4) | -18.6  (-51.7-14.5) | 2.8  (-57.9-63.6) |
|  | Yorkshire and The Humber | -17.1  (-39.4-5.1) | 6.0  (-10.4-22.5) | 7.1  (-5.2-19.5) | 1.7  (-7.1-10.5) | 6.0  (-4.4-16.4) | 15.9*  (2.0-29.8) | 16.7*  (0.8-32.6) | 18.9*  (0.7-37.1) | 21.6  (-0.1-43.3) | 14.1  (-14.3-42.4) | 16.1  (-33.4-65.7) |
| **Sex** | Females | 12.1*  (2.6-21.7) | 7.4*  (0.4-14.5) | 9.0**  (3.8-14.2) | 3.8*  (0.1-7.5) | 4.4*  (0.1-8.7) | 15.7**  (9.9-21.5) | 15.9** (9.3-22.5) | 14.3**  (6.8-21.8) | 13.0**  (4.1-21.9) | 11.7*  (0.2-23.1) | 18.5  (-1.9-38.9) |
|  | Males | -21.6*  (-38.5--4.7) | -11.5  (-24.1-1.0) | -4.6  (-13.8-4.6) | -2.2  (-8.9-4.5) | 9.4*  (1.3-17.5) | 23.9**  (12.9-34.9) | 24.8** (12.4-37.2) | 22.7**  (8.8-36.6) | 23.8**  (7.4-40.2) | 16.4  (-4.7-37.5) | 2.2  (-36.4-40.7) |
| **Therapy intensity** | High intensity | -1.7  (-12.8-9.3) | -4.8  (-13.0-3.4) | 0.7  (-5.3-6.7) | 1.1  (-3.3-5.4) | 3.4  (-1.7-8.4) | 17.5**  (10.7-24.3) | 19.8** (12.1-27.5) | 16.5**  (7.8-25.2) | 16.0**  (5.7-26.2) | 11.3  (-1.8-24.4) | 2.9  (-21.1-26.8) |
|  | Low intensity | 11.2  (-1.9-24.4) | 14.9**  (5.3-24.6) | 14.7**  (7.5-21.8) | 5.5*  (0.4-10.7) | 7.8*  (1.5-14.0) | 15.3**  (6.9-23.8) | 11.9* (2.3-21.4) | 12.1*  (1.2-22.9) | 10.2  (-2.8-23.1) | 8.2  (-8.7-25.1) | 22.3  (-7.6-52.2) |
|  | Missing | -36.0  (-199.3-127.3) | -23.4  (-154.8-108.0) | -45.5  (-142.6-51.7) | -5.2  (-77.7-67.2) | -15.4  (-101.4-70.7) | 25.9  (-91.4-143.3) | -27.4 (-165.9-111.0) | -45.7  (-209.7-118.2) | 5.7  (-191.3-202.8) | -94.2  (-330.9-142.5) | -235.4  (-572.5-101.7) |

Notes:

1. * = p value <0.05 and ** = p value <0.01
2. The dotted line marks the intervention point; data to the left of the line correspond to the pre-intervention period, while data to the right reflect the post-intervention period.
3. Deprivation = The dimensions of deprivation used to classify households are indicators based on four selected household characteristics: education, employment, health and housing.
4. NS-SEC = National Statistics Socio-Economic Classification.
5. All reported confidence intervals around point estimates are at the 95% level and are estimated using robust standard errors clustered at an individual level.

# **Supplementary Table 7.** Effects of NHS TT treatment completion on probability of being a paid employee broken-down by socio-demographic and mental health related characteristics.

| **Characteristic** | **Category** | **Time since first therapy in years** | | | | | | | | | | |
| --- | --- | --- | --- | --- | --- | --- | --- | --- | --- | --- | --- | --- |
|  |  | **-4** | **-3** | **-2** | **-1** | **1** | **2** | **3** | **4** | **5** | **6** | **7** |
| **Age band** | 25-34 | 0.6* (0.1-1.0) | 0.6** (0.2-0.9) | 0.5** (0.2-0.7) | 0.2* (0.0-0.4) | 0.7** (0.5-0.9) | 1.5** (1.2-1.8) | 1.8** (1.6-2.1) | 1.9** (1.6-2.2) | 1.7** (1.3-2.1) | 1.9** (1.4-2.3) | 2.3** (1.4-3.2) |
|  | 35-44 | 0.4 (-0.1-0.9) | 0.2 (-0.1-0.6) | 0.2 (0.0-0.5) | 0.1 (-0.1-0.3) | 0.6** (0.3-0.8) | 1.3** (1.0-1.6) | 1.7** (1.4-2.0) | 1.9** (1.5-2.2) | 2.0** (1.6-2.4) | 2.0** (1.5-2.5) | 1.9** (0.9-2.8) |
|  | 45-54 | 0.6* (0.1-1.1) | 0.3 (0.0-0.7) | 0.2 (-0.1-0.5) | 0.0 (-0.2-0.2) | 0.4** (0.1-0.6) | 0.8** (0.5-1.2) | 1.0** (0.6-1.3) | 1.0** (0.6-1.4) | 0.9** (0.4-1.4) | 0.7* (0.1-1.3) | 1.0 (0.0-2.0) |
|  | 55-60 | 0.3 (-0.5-1.1) | 0.1 (-0.5-0.6) | 0.0 (-0.4-0.4) | 0.0 (-0.3-0.3) | 0.1 (-0.3-0.5) | 0.2 (-0.4-0.7) | 0.0 (-0.6-0.6) | -0.3 (-1.0-0.4) | -0.7 (-1.6-0.1) | -1.2* (-2.3--0.1) | -1.5 (-3.6-0.6) |
| **Deprivation** | Deprived, 1 dimension | 0.5 (0.0-0.9) | 0.5** (0.1-0.9) | 0.3 (0.0-0.5) | 0.0 (-0.2-0.2) | 0.6** (0.4-0.8) | 1.4** (1.1-1.7) | 1.7** (1.4-2.1) | 1.6** (1.2-2.0) | 1.6** (1.1-2.0) | 1.5** (1.0-2.1) | 2.1** (1.1-3.1) |
|  | Deprived, 2 dimensions | 1.0** (0.3-1.7) | 0.7** (0.2-1.2) | 0.4* (0.0-0.8) | 0.2 (-0.1-0.5) | 0.2 (-0.1-0.5) | 1.0** (0.6-1.4) | 1.2** (0.8-1.7) | 1.4** (0.9-1.9) | 1.2** (0.6-1.8) | 1.4** (0.6-2.1) | 1.7* (0.4-3.0) |
|  | Deprived, 3 dimensions | 0.2 (-0.8-1.3) | 0.4 (-0.4-1.1) | 0.4 (-0.2-1.0) | -0.1 (-0.5-0.3) | 0.4 (0.0-0.9) | 1.0** (0.4-1.6) | 1.0** (0.4-1.7) | 1.2** (0.4-1.9) | 1.3** (0.5-2.1) | 1.3* (0.3-2.4) | 2.3* (0.4-4.2) |
|  | Deprived, 4 dimensions | 2.1 (-1.1-5.3) | 1.1 (-1.1-3.4) | 0.5 (-1.2-2.2) | -0.1 (-1.3-1.1) | -0.3 (-1.6-1.0) | 0.3 (-1.5-2.1) | 1.5 (-0.5-3.5) | 1.7 (-0.5-3.8) | 2.4* (0.0-4.9) | 2.8 (-0.4-6.0) | 5.1 (-0.8-11.0) |
|  | Not deprived | 0.4* (0.1-0.8) | 0.2 (-0.1-0.5) | 0.3** (0.1-0.5) | 0.1 (0.0-0.3) | 0.5** (0.4-0.7) | 1.0** (0.8-1.3) | 1.2** (1.0-1.5) | 1.3** (1.0-1.5) | 1.2** (0.8-1.5) | 1.1** (0.6-1.5) | 0.9* (0.1-1.6) |
| **Employment status** | Employed | 0.7** (0.4-1.0) | 0.6** (0.4-0.8) | 0.5** (0.3-0.7) | 0.2** (0.1-0.4) | 0.7** (0.6-0.8) | 1.2** (1.0-1.4) | 1.4** (1.2-1.6) | 1.4** (1.1-1.6) | 1.3** (1.0-1.6) | 1.3** (0.9-1.6) | 1.2** (0.5-1.9) |
|  | Not working, long-term sick/disabled | 0.6 (-0.3-1.6) | -0.1 (-0.8-0.6) | -0.5 (-1.0-0.1) | -0.6** (-1.0--0.1) | -0.5* (-0.9-0.0) | -0.1 (-0.6-0.4) | 0.1 (-0.5-0.6) | 0.2 (-0.4-0.8) | 0.2 (-0.5-0.8) | 0.1 (-0.7-1.0) | 0.6 (-0.8-2.1) |
|  | Student | 1.1 (-2.1-4.2) | 0.9 (-1.5-3.3) | 0.2 (-1.8-2.1) | 0.1 (-1.5-1.6) | -0.2 (-1.9-1.4) | 0.7 (-1.3-2.8) | 1.2 (-0.9-3.4) | 1.9 (-0.4-4.2) | 0.8 (-1.8-3.4) | 2.0 (-1.2-5.1) | 3.9 (-1.7-9.6) |
|  | Not working, not seeking work | 1.4* (0.2-2.5) | 0.7 (-0.1-1.6) | 0.7* (0.0-1.3) | 0.0 (-0.5-0.4) | -0.1 (-0.6-0.4) | 1.1** (0.5-1.8) | 1.3** (0.6-2.0) | 1.5** (0.7-2.3) | 1.3** (0.4-2.2) | 1.3* (0.1-2.4) | 0.5 (-1.5-2.5) |
|  | Not working, seeking work | 1.4** (0.4-2.4) | 0.8 (0.0-1.6) | 0.7* (0.1-1.4) | 0.4 (-0.1-0.9) | 0.7* (0.2-1.3) | 2.3** (1.6-2.9) | 2.9** (2.2-3.5) | 3.1** (2.4-3.9) | 3.1** (2.3-3.9) | 2.9** (1.9-4.0) | 3.0** (1.3-4.7) |
|  | Missing or not stated | -1.1** (-2.0--0.3) | -0.9** (-1.6--0.3) | -0.8** (-1.2--0.3) | -0.4* (-0.7-0.0) | 0.2 (-0.1-0.6) | 0.6* (0.1-1.1) | 0.8** (0.2-1.4) | 0.9** (0.3-1.5) | 0.6 (-0.1-1.4) | 0.4 (-0.5-1.3) | 2.8** (1.0-4.5) |
| **Ethnicity** | Asian | -0.8 (-2.0-0.4) | -0.5 (-1.5-0.4) | 0.0 (-0.7-0.7) | -0.1 (-0.6-0.4) | 0.6* (0.0-1.2) | 1.6** (0.8-2.3) | 1.7** (0.8-2.5) | 1.3** (0.4-2.2) | 1.1* (0.0-2.2) | 1.4* (0.0-2.8) | 2.0 (-0.6-4.5) |
|  | Black | -0.3 (-2.0-1.4) | 0.2 (-1.1-1.4) | 0.2 (-0.8-1.2) | -0.2 (-0.9-0.6) | 0.0 (-0.8-0.8) | 0.3 (-0.8-1.4) | 0.3 (-0.9-1.5) | 0.9 (-0.4-2.2) | 0.2 (-1.4-1.7) | 0.7 (-1.2-2.7) | 1.7 (-1.7-5.2) |
|  | Mixed | -0.5 (-2.4-1.4) | -0.2 (-1.6-1.2) | 0.2 (-0.9-1.3) | -0.7 (-1.5-0.1) | -0.2 (-1.1-0.7) | 0.3 (-0.8-1.5) | 0.7 (-0.6-2.0) | 0.3 (-1.1-1.7) | 0.1 (-1.5-1.8) | 1.4 (-0.7-3.6) | 2.9 (-0.9-6.7) |
|  | Other | -0.6 (-4.1-2.9) | -0.2 (-2.7-2.4) | -0.2 (-2.1-1.7) | 0.0 (-1.4-1.4) | 0.6 (-0.9-2.1) | 1.7 (-0.3-3.7) | 1.4 (-0.9-3.7) | 1.2 (-1.3-3.7) | 2.9* (0.0-5.8) | 4.1* (0.3-7.9) | 3.6 (-3.6-10.7) |
|  | White | 0.7** (0.4-0.9) | 0.5** (0.3-0.7) | 0.3** (0.2-0.5) | 0.1** (0.0-0.3) | 0.5** (0.4-0.6) | 1.1** (1.0-1.3) | 1.4** (1.2-1.6) | 1.5** (1.3-1.7) | 1.4** (1.2-1.7) | 1.3** (1.0-1.6) | 1.4** (0.9-2.0) |
| **Highest qualification** | Apprenticeship | -0.8 (-2.7-1.1) | -0.3 (-1.7-1.1) | -0.6 (-1.7-0.4) | -0.4 (-1.1-0.4) | 0.2 (-0.7-1.1) | 0.3 (-0.9-1.5) | 0.7 (-0.7-2.1) | 1.1 (-0.5-2.7) | 1.1 (-0.8-3.0) | 1.8 (-0.7-4.3) | 3.3 (-1.0-7.6) |
|  | Level 1 | 0.9** (0.2-1.5) | 0.5* (0.0-1.0) | 0.2 (-0.1-0.6) | 0.2 (0.0-0.5) | 0.5** (0.2-0.8) | 1.2** (0.8-1.6) | 1.5** (1.0-1.9) | 1.5** (1.0-1.9) | 1.2** (0.6-1.8) | 1.4** (0.6-2.1) | 1.4* (0.1-2.7) |
|  | Level 2 | 0.1 (-0.5-0.7) | 0.1 (-0.4-0.5) | 0.2 (-0.1-0.6) | 0.0 (-0.3-0.2) | 0.5** (0.2-0.8) | 1.3** (1.0-1.7) | 1.5** (1.1-1.9) | 1.5** (1.1-1.9) | 1.3** (0.8-1.8) | 1.1** (0.4-1.8) | 1.9** (0.6-3.1) |
|  | Level 3 | 0.9** (0.3-1.5) | 0.4* (0.0-0.9) | 0.3 (0.0-0.6) | 0.1 (-0.2-0.3) | 0.6** (0.3-0.9) | 1.5** (1.1-1.8) | 1.8** (1.4-2.2) | 1.9** (1.4-2.3) | 1.8** (1.3-2.3) | 1.7** (1.0-2.3) | 1.2 (-0.1-2.4) |
|  | Level 4 and above | 0.6* (0.1-1.0) | 0.5** (0.1-0.8) | 0.3** (0.1-0.6) | 0.2* (0.0-0.4) | 0.4** (0.2-0.6) | 0.8** (0.5-1.1) | 1.0** (0.7-1.3) | 1.0** (0.6-1.3) | 1.1** (0.6-1.5) | 1.1** (0.5-1.6) | 1.2* (0.2-2.1) |
|  | No qualifications | 1.2** (0.4-2.0) | 1.1** (0.5-1.7) | 0.7** (0.3-1.2) | 0.0 (-0.3-0.3) | 0.6** (0.2-1.0) | 1.0** (0.5-1.5) | 1.3** (0.8-1.8) | 1.4** (0.8-2.0) | 1.3** (0.6-2.0) | 1.0* (0.1-1.9) | 1.4 (-0.1-2.9) |
|  | Other | -1.1 (-2.8-0.6) | -0.4 (-1.7-0.8) | 0.3 (-0.6-1.2) | 0.2 (-0.5-0.8) | 0.2 (-0.5-0.9) | 0.8 (-0.2-1.7) | 1.2* (0.1-2.3) | 1.6* (0.3-2.8) | 1.7* (0.3-3.2) | 2.4** (0.6-4.3) | 2.8 (-0.3-6.0) |
| **Medication** | Not prescribed | 0.5* (0.1-0.9) | 0.4** (0.1-0.7) | 0.4** (0.1-0.6) | 0.1 (0.0-0.3) | 0.3** (0.2-0.5) | 0.9** (0.7-1.2) | 1.3** (1.0-1.6) | 1.4** (1.1-1.7) | 1.4** (1.1-1.8) | 1.5** (1.0-2.0) | 1.8** (0.9-2.7) |
|  | Prescribed, not taking | 0.5 (-0.7-1.7) | 0.8 (-0.1-1.7) | 0.8* (0.1-1.5) | 0.3 (-0.1-0.8) | 0.3 (-0.3-0.8) | 1.1** (0.3-1.8) | 1.0* (0.2-1.9) | 0.8 (-0.1-1.8) | 0.7 (-0.4-1.7) | 1.2 (-0.1-2.6) | 2.7* (0.4-5.0) |
|  | Prescribed, taking | 0.7** (0.3-1.0) | 0.4** (0.1-0.6) | 0.3** (0.1-0.5) | 0.1 (0.0-0.2) | 0.6** (0.5-0.8) | 1.3** (1.1-1.6) | 1.5** (1.3-1.8) | 1.5** (1.2-1.8) | 1.4** (1.1-1.8) | 1.4** (1.0-1.8) | 1.4** (0.6-2.1) |
|  | Missing or not stated | -0.1 (-1.3-1.0) | 0.1 (-0.8-1.0) | -0.3 (-1.0-0.3) | -0.2 (-0.7-0.2) | 0.7** (0.2-1.2) | 1.1** (0.4-1.8) | 1.1** (0.3-1.9) | 1.0* (0.1-1.9) | 0.2 (-0.8-1.3) | -0.2 (-1.5-1.0) | -0.6 (-2.8-1.6) |
| **NS-SEC** | Full-Time students | -0.5 (-1.3-0.3) | -0.5 (-1.1-0.2) | 0.3 (-0.2-0.8) | 0.0 (-0.3-0.4) | 0.6** (0.3-1.0) | 1.5** (1.0-2.0) | 1.9** (1.4-2.5) | 2.0** (1.3-2.6) | 2.0** (1.3-2.8) | 2.2** (1.1-3.3) | 3.1** (1.0-5.2) |
|  | Higher managerial, administrative and professional | 0.3 (-0.6-1.3) | 0.1 (-0.6-0.8) | 0.2 (-0.3-0.7) | 0.2 (-0.2-0.6) | 0.7** (0.2-1.1) | 1.3** (0.7-1.9) | 1.3** (0.6-2.0) | 1.1** (0.4-1.8) | 1.1* (0.2-2.0) | 0.5 (-0.6-1.5) | 0.6 (-1.3-2.5) |
|  | Intermediate occupations | 1.0** (0.4-1.6) | 0.7** (0.3-1.2) | 0.5** (0.1-0.8) | 0.2 (0.0-0.4) | 0.6** (0.3-0.9) | 1.2** (0.8-1.6) | 1.4** (0.9-1.8) | 1.4** (0.9-1.9) | 1.0** (0.4-1.6) | 0.8* (0.1-1.5) | 1.3* (0.1-2.6) |
|  | Long-term unemployed | 0.6 (-1.3-2.4) | -0.3 (-1.6-1.1) | -0.2 (-1.3-0.8) | 0.0 (-0.8-0.7) | -0.1 (-0.9-0.8) | 0.7 (-0.3-1.8) | 0.9 (-0.3-2.1) | 1.2 (0.0-2.5) | 1.5* (0.0-2.9) | 0.9 (-0.9-2.7) | 0.2 (-3.1-3.5) |
|  | Lower managerial, administrative and professional | 0.4 (-0.1-0.9) | 0.3 (-0.1-0.7) | 0.3 (0.0-0.6) | 0.1 (-0.1-0.3) | 0.3** (0.1-0.6) | 0.8** (0.5-1.2) | 1.0** (0.6-1.4) | 1.0** (0.5-1.4) | 1.0** (0.5-1.5) | 1.1** (0.5-1.7) | 1.3* (0.2-2.4) |
|  | Lower supervisory and technical occupations | 0.6 (-0.5-1.6) | 0.7 (0.0-1.5) | 0.0 (-0.6-0.6) | 0.1 (-0.4-0.5) | 0.8** (0.4-1.3) | 1.5** (0.9-2.2) | 1.8** (1.1-2.5) | 1.7** (0.9-2.5) | 1.6** (0.7-2.6) | 1.8** (0.6-3.0) | 0.8 (-1.3-2.9) |
|  | Never worked | -0.2 (-1.4-1.1) | 0.0 (-0.9-1.0) | -0.4 (-1.1-0.4) | -0.2 (-0.7-0.3) | 0.4 (-0.2-0.9) | 1.0** (0.3-1.7) | 1.1** (0.3-1.9) | 1.2** (0.4-2.1) | 1.2* (0.2-2.3) | 1.4* (0.1-2.7) | 2.0 (-0.5-4.4) |
|  | Routine occupations | 2.0** (1.1-2.9) | 1.5** (0.8-2.2) | 0.8** (0.3-1.3) | 0.3 (0.0-0.7) | 0.5* (0.1-0.9) | 1.2** (0.7-1.7) | 1.4** (0.8-2.0) | 1.7** (1.0-2.4) | 1.5** (0.7-2.3) | 1.3** (0.4-2.3) | 1.9* (0.2-3.7) |
|  | Semi-routine occupations | 1.4** (0.8-2.0) | 0.9** (0.4-1.4) | 0.6** (0.2-0.9) | 0.2 (-0.1-0.5) | 0.4** (0.1-0.7) | 1.2** (0.8-1.6) | 1.7** (1.3-2.2) | 1.8** (1.3-2.3) | 1.9** (1.3-2.4) | 2.0** (1.3-2.7) | 1.4* (0.1-2.7) |
|  | Small employers and own account workers | -1.6* (-2.9--0.3) | -0.9 (-1.8-0.0) | -0.4 (-1.1-0.3) | -0.6* (-1.1--0.1) | 0.5 (0.0-1.1) | 0.8* (0.1-1.5) | 0.9* (0.1-1.7) | 1.0* (0.1-1.9) | 0.7 (-0.4-1.8) | 1.0 (-0.3-2.4) | 2.3 (-0.2-4.7) |
| **Number of treatment sessions** | 2 | 0.3 (-0.4-1.0) | 0.1 (-0.5-0.6) | -0.1 (-0.5-0.3) | -0.2 (-0.5-0.1) | 0.6** (0.3-0.9) | 0.8** (0.3-1.2) | 0.9** (0.4-1.4) | 1.1** (0.5-1.6) | 0.5 (-0.1-1.2) | -0.1 (-1.0-0.7) | -0.3 (-1.8-1.3) |
|  | 3 to 5 | 0.5* (0.1-0.9) | 0.4* (0.1-0.7) | 0.2 (0-0.4) | 0 (-0.2-0.2) | 0.8** (0.6-1.0) | 1.2** (1.0-1.5) | 1.5** (1.2-1.8) | 1.5** (1.1-1.8) | 1.4** (1.0-1.8) | 1.5** (1.0-2.0) | 2.0** (1.1-2.9) |
|  | 6 to 8 | 0.8** (0.2-1.4) | 0.6** (0.2-1.1) | 0.5** (0.2-0.9) | 0.2 (-0.1-0.4) | 1.0** (0.7-1.3) | 1.9** (1.5-2.3) | 1.9** (1.5-2.3) | 1.9** (1.4-2.4) | 2.0** (1.4-2.5) | 1.6** (0.9-2.3) | 2.0** (0.7-3.3) |
|  | 9 to 15 | 0.4 (-0.5-1.2) | -0.3 (-1.0-0.3) | 0 (-0.5-0.5) | -0.1 (-0.5-0.2) | 0.8** (0.4-1.2) | 1.4** (0.9-1.9) | 1.6** (1.0-2.2) | 1.5** (0.8-2.2) | 0.9* (0.1-1.7) | 1.0 (0-2.0) | 0.2 (-1.6-2.0) |
|  | 16 or more | 1.6 (-0.5-3.8) | 0.3 (-1.2-1.8) | 0.2 (-0.9-1.3) | 0.4 (-0.3-1.2) | 0.7 (-0.2-1.5) | 1.8** (0.6-3.1) | 1.6* (0.2-3.0) | 1.2 (-0.3-2.7) | 1.3 (-0.5-3.0) | 1.2 (-0.9-3.3) | -0.7 (-3.8-2.4) |
| **Region** | East Midlands | 0.7 (-0.2-1.5) | 0.5 (-0.2-1.1) | 0.4 (-0.1-0.9) | 0.2 (-0.2-0.5) | 0.5* (0.1-0.9) | 0.8** (0.3-1.4) | 1.0** (0.3-1.6) | 0.9* (0.2-1.5) | 1.4** (0.6-2.3) | 1.4** (0.4-2.5) | 1.8 (-0.2-3.7) |
|  | East of England | 1.2** (0.4-2.1) | 0.8* (0.2-1.4) | 0.4 (-0.1-0.8) | 0.0 (-0.4-0.3) | -0.2 (-0.5-0.2) | 0.8** (0.3-1.2) | 1.2** (0.6-1.7) | 1.4** (0.7-2.0) | 1.1** (0.4-1.8) | 1.2* (0.3-2.1) | 2.0* (0.4-3.7) |
|  | London | 0.1 (-0.6-0.8) | 0.2 (-0.3-0.7) | 0.2 (-0.2-0.6) | 0.2 (-0.1-0.5) | 0.6** (0.3-1.0) | 1.4** (1.0-1.9) | 1.7** (1.2-2.1) | 1.7** (1.2-2.3) | 1.8** (1.2-2.5) | 2.3** (1.5-3.1) | 2.6** (1.1-4.1) |
|  | North East | 0.9 (-0.1-1.9) | 0.7* (0.0-1.4) | 0.4 (-0.2-0.9) | 0.1 (-0.2-0.5) | 0.4 (0.0-0.9) | 1.1** (0.5-1.7) | 1.5** (0.8-2.1) | 1.4** (0.7-2.2) | 1.4** (0.5-2.2) | 0.7 (-0.4-1.8) | 0.4 (-1.4-2.2) |
|  | North West | 0.3 (-0.4-0.9) | 0.6* (0.1-1.0) | 0.5** (0.1-0.8) | 0.1 (-0.1-0.4) | 0.8** (0.5-1.1) | 1.4** (1.0-1.8) | 1.5** (1.1-2.0) | 1.6** (1.1-2.1) | 1.6** (1.0-2.2) | 1.8** (1.0-2.6) | 2.1** (0.7-3.6) |
|  | South East | 0.7 (0.0-1.4) | 0.2 (-0.3-0.7) | 0.2 (-0.2-0.6) | 0.2 (-0.1-0.4) | 0.6** (0.3-0.9) | 1.4** (1.0-1.8) | 1.7** (1.2-2.1) | 1.6** (1.1-2.1) | 1.5** (0.9-2.2) | 1.7** (0.9-2.5) | 1.7* (0.3-3.2) |
|  | South West | -0.1 (-0.9-0.8) | 0.1 (-0.6-0.7) | 0.0 (-0.5-0.5) | -0.1 (-0.4-0.3) | 0.3 (-0.1-0.7) | 0.6* (0.1-1.1) | 0.8** (0.3-1.4) | 0.8* (0.1-1.4) | 0.4 (-0.4-1.1) | 0.8 (-0.2-1.8) | 1.1 (-0.6-2.8) |
|  | West Midlands | 1.0* (0.2-1.8) | 0.5 (-0.1-1.1) | 0.5 (0.0-0.9) | 0.2 (-0.1-0.5) | 0.8** (0.4-1.2) | 1.5** (1.0-2.0) | 1.6** (1.0-2.2) | 1.8** (1.2-2.5) | 1.1** (0.3-1.9) | 0.4 (-0.6-1.4) | 0.5 (-1.3-2.3) |
|  | Yorkshire and The Humber | 0.2 (-0.5-1.0) | 0.1 (-0.5-0.6) | 0.1 (-0.3-0.5) | 0.0 (-0.3-0.3) | 0.5** (0.2-0.9) | 1.1** (0.6-1.6) | 1.4** (0.9-2.0) | 1.5** (0.9-2.1) | 1.7** (1.0-2.4) | 0.9* (0.0-1.8) | 0.5 (-1.0-2.1) |
| **Sex** | Females | 0.9** (0.5-1.2) | 0.6** (0.4-0.8) | 0.4** (0.3-0.6) | 0.2* (0.0-0.3) | 0.5** (0.4-0.7) | 1.2** (1.0-1.4) | 1.4** (1.2-1.6) | 1.4** (1.2-1.7) | 1.4** (1.1-1.7) | 1.3** (1.0-1.7) | 1.5** (0.9-2.2) |
|  | Males | -0.3 (-0.7-0.1) | -0.2 (-0.5-0.2) | -0.1 (-0.3-0.2) | 0.0 (-0.2-0.1) | 0.5** (0.3-0.7) | 1.1** (0.8-1.4) | 1.3** (1.0-1.7) | 1.4** (1.1-1.8) | 1.3** (0.9-1.7) | 1.3** (0.8-1.8) | 1.4** (0.5-2.3) |
| **Diagnosis** | Agoraphobia | -0.2 (-3.4-3.0) | 1.3 (-1.0-3.7) | 0.7 (-1.1-2.5) | 0.0 (-1.3-1.3) | 0.5 (-0.9-1.9) | 1.3 (-0.6-3.1) | 1.8 (-0.2-3.9) | 0.9 (-1.3-3.2) | 2.6 (-0.1-5.3) | 3.9* (0.3-7.5) | 2.4 (-3.9-8.8) |
|  | Depression | 0.6** (0.2-1.0) | 0.4** (0.1-0.7) | 0.4** (0.1-0.6) | 0.1 (-0.1-0.2) | 0.7** (0.5-0.9) | 1.4** (1.1-1.6) | 1.7** (1.4-2.0) | 1.6** (1.3-1.9) | 1.5** (1.1-1.9) | 1.6** (1.1-2.1) | 1.8** (0.8-2.8) |
|  | Generalized anxiety disorder | 0.6* (0.1-1.1) | 0.5* (0.1-0.8) | 0.4** (0.1-0.7) | 0.1 (-0.1-0.3) | 0.4** (0.2-0.6) | 0.9** (0.6-1.3) | 1.0** (0.7-1.4) | 1.2** (0.8-1.6) | 1.0** (0.5-1.4) | 0.7* (0.1-1.4) | 0.8 (-0.4-2.0) |
|  | Mixed anxiety and depressive disorder | -0.3 (-1.4-0.9) | -0.2 (-0.9-0.6) | -0.1 (-0.6-0.4) | -0.1 (-0.5-0.2) | 0.4* (0.0-0.8) | 1.1** (0.6-1.6) | 1.4** (0.8-2.0) | 1.6** (0.9-2.2) | 1.5** (0.8-2.2) | 1.3** (0.5-2.2) | 1.8** (0.5-3.0) |
|  | Obsessive-compulsive disorder | 1.1 (-0.7-3.0) | 0.7 (-0.7-2.2) | 0.8 (-0.3-1.8) | 0.7 (0.0-1.5) | 0.4 (-0.5-1.2) | 0.1 (-1.0-1.2) | 1.0 (-0.3-2.2) | 0.9 (-0.5-2.4) | 1.2 (-0.4-2.8) | 2.3* (0.1-4.4) | 0.8 (-3.1-4.8) |
|  | Other anxiety or stress related disorder | 0.7 (-0.6-2.0) | 0.9 (-0.1-1.8) | 0.3 (-0.5-1.0) | 0.3 (-0.2-0.9) | 0.8** (0.2-1.4) | 1.1** (0.3-1.9) | 1.4** (0.4-2.3) | 1.4** (0.4-2.4) | 1.4* (0.2-2.7) | 1.5 (-0.2-3.1) | 0.6 (-2.4-3.7) |
|  | Panic disorder | 0.3 (-1.4-2.0) | -0.1 (-1.3-1.1) | -0.7 (-1.5-0.2) | 0.2 (-0.4-0.8) | 0.9* (0.2-1.6) | 1.8** (0.8-2.7) | 1.8** (0.7-2.9) | 1.8** (0.6-3.0) | 2.4** (1.0-3.8) | 2.1* (0.3-3.9) | 1.2 (-2.1-4.5) |
|  | Post-traumatic stress disorder | 0.8 (-0.6-2.1) | 0.1 (-1.0-1.2) | 0.3 (-0.5-1.1) | -0.2 (-0.8-0.4) | 0.0 (-0.7-0.6) | 0.8 (-0.1-1.7) | 1.0 (0.0-2.0) | 1.2* (0.2-2.3) | 0.9 (-0.4-2.2) | -0.3 (-2.0-1.4) | -0.3 (-3.4-2.9) |
|  | Social phobias | 1.8* (0.0-3.6) | 0.5 (-0.9-1.8) | 0.3 (-0.8-1.3) | 0.0 (-0.8-0.7) | 0.4 (-0.4-1.2) | 1.6** (0.5-2.7) | 1.3* (0.0-2.5) | 1.8** (0.5-3.1) | 1.3 (-0.3-2.9) | 1.0 (-1.0-3.1) | -0.5 (-4.5-3.5) |
|  | Specific phobias | 2.0 (-1.1-5.0) | 1.0 (-1.3-3.2) | 0.6 (-1.0-2.2) | 0.0 (-1.2-1.2) | -0.3 (-1.6-1.0) | -0.2 (-2.0-1.6) | 1.3 (-0.8-3.4) | 0.8 (-1.5-3.2) | 0.1 (-2.7-2.8) | 0.1 (-3.5-3.8) | 3.7 (-2.5-9.9) |
|  | Missing or not stated | 0.2 (-0.6-1.1) | 0.3 (-0.3-0.9) | 0.4 (0.0-0.8) | 0.3 (0.0-0.6) | 0.2 (-0.1-0.5) | 0.9** (0.4-1.3) | 1.1** (0.6-1.6) | 1.1** (0.5-1.6) | 1.0** (0.4-1.7) | 1.2** (0.4-2.0) | 1.6* (0.4-2.9) |
| **Therapy intensity** | High intensity | 0.4* (0.1-0.7) | 0.2 (-0.1-0.5) | 0.2* (0.0-0.4) | 0.1 (-0.1-0.2) | 0.4** (0.2-0.6) | 1.0** (0.8-1.3) | 1.3** (1.1-1.6) | 1.3** (1.0-1.5) | 1.2** (0.9-1.5) | 1.3** (0.9-1.7) | 1.2** (0.5-1.9) |
|  | Low intensity | 0.8** (0.4-1.2) | 0.7** (0.4-1.0) | 0.4** (0.2-0.7) | 0.1 (0.0-0.3) | 0.7** (0.5-0.8) | 1.3** (1.0-1.5) | 1.5** (1.2-1.8) | 1.7** (1.4-2.0) | 1.5** (1.2-1.9) | 1.4** (0.9-1.8) | 2.0** (1.2-2.9) |
|  | Missing | 0.8 (-3.3-4.9) | -0.1 (-3.6-3.4) | -1.6 (-4.7-1.5) | -0.4 (-2.6-1.8) | 0.5 (-1.8-2.7) | 2.3 (-0.9-5.5) | 0.5 (-3.1-4.1) | -1.8 (-6.2-2.5) | 0.4 (-5.4-6.1) | 1.8 (-6.0-9.6) | -1.1 (-10.9-8.6) |

Notes:

1. * = p value <0.05 and ** = p value <0.01
2. The dotted line marks the intervention point; data to the left of the line correspond to the pre-intervention period, while data to the right reflect the post-intervention period.
3. Deprivation = The dimensions of deprivation used to classify households are indicators based on four selected household characteristics: education, employment, health and housing.
4. NS-SEC = National Statistics Socio-Economic Classification.
5. All reported confidence intervals around point estimates are at the 95% level and are estimated using robust standard errors clustered at an individual level.

# **Supplementary Appendix A:** Heterogenous treatment effects

Methods

We also looked at heterogenous treatment effects by sociodemographic characteristics and labour market status, before applying a Generalized Random Forest (GRF) approach^1^ to study treatment heterogeneity effects without pre-selecting its sources. To adapt the model for GRF, we made two adjustments. First, since GRF estimates a single effect at a time, we focused on a single year. Year four was selected post-treatment to balance an ample follow-up period with a sufficient sample size. This subsample includes 630,991 observations, representing 75.0% of the total sample in the main analysis.

Next, as the GRF model does not incorporate covariates in the same way as traditional regression, we did not include any individual fixed effects. We transformed the outcome to reflect the change from the average level one year before treatment to the average four years after, to maintain a within-person estimate. The parameter of interest is presented in equation (2).


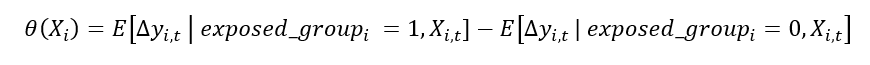
(2)

where $\Delta y_{i,t}=(\bar{y}_{i,y=year4}-\bar{y}_{i,y=year0})$ is a change in the outcome of an individual *i* who had their first therapy session at time t, and is a set of covariates.

The GRF creates multiple ‘decision trees,’ each of which sequentially splits the data into bins of observations that are similar within the bin but distinct from other bins in terms of the treatment effect. The treatment effect is then estimated for each bin. By sampling random subsets of data to build each tree and averaging the estimates for each individual across trees, GRF avoids overfitting and captures general patterns rather than any single tree’s result. In practice, the algorithm also uses different subsamples for growing trees and treatment effect estimation. This is known as the honest approach and helps reduce the risks of overfitting and biased estimates^24^. A histogram of individual estimates illustrates the range and variability of effects across the population. The algorithm also provides an overall average treatment effect.

In GRF, each decision tree split is based on a covariate, e.g. age being above or below a threshold. The *variable importance* *score* helps identify which covariates are most related to the differences in treatment effects by tracking how frequently each variable is used for splits, with greater weight given to splits earlier in the tree.

Like the parametric model above, the GRF accounts for the fact that this analysis, both the outcome and exposure likelihood can be partially explained by covariates. This was done by ‘orthogonalizing’ the forest: we first estimated individual propensity scores and outcomes using regression forests. We then calculated the residuals for the exposure and outcome variables and estimate a causal forest on these residuals. See Nie and Wager (2021) for details^2^.

The GRF algorithm was implemented with the grf package in R. The results are presented for 2,000 trees. To assess the sensitivity of the results, parts of the analysis were replicated for 500 trees. The results remained qualitatively similar.

Results

Using the GRF approach, for both outcomes, monthly earnings and probability of being a paid employee, we found heterogeneity for respondents with different characteristics four years after therapy. Neither of the two estimated distributions was bounded away from zero, implying that there are groups of patients who do not benefit from treatment (**Supplementary Appendix A: Figure 1**).

**Supplementary Appendix A: Figure 1.** Distribution of predicted treatment effects for (A) monthly earning and (B) probability of being a paid employee.


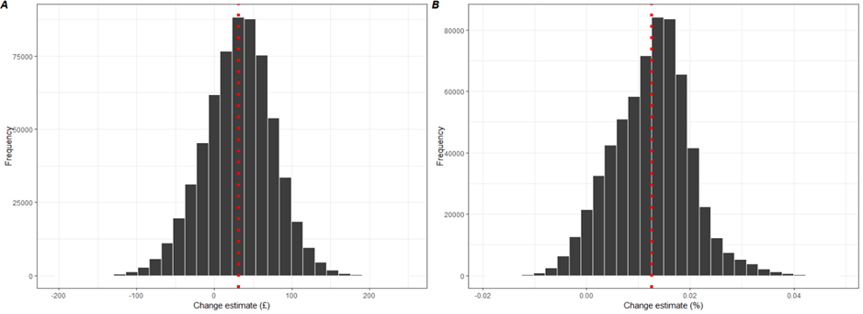


This is supported by results from **Figure.3A**. in the main paper which show that the main benefits of NHSTT are in population ‘Not working, seeking work’.

The conditional average treatment effect on the treated group was £23.2 (s.e.=12.0) for pay and 1.1 p.p. for probability of being a paid employee (s.e.=0.1). The top three variables identified as important for heterogeneity in probability of being a paid employee were age band (18.2%), ‘Not working, seeking work’ (14.3%) and ‘Students’ (10.2%). For monthly earnings, the top three variables were age bands (6.7%), quarter of first therapy (3.4%) and dependent children (3.1%). The importance of other variables can be found in **Supplementary Appendix A: Table 1**. Additionally, when restricting the population across the study period to individuals in paid employment only (pay>0), the results from the fitted linear regression model with individual fixed effects found negative effects on monthly earnings of maximum of -£27.8 in year seven relative to one year pre-treatment (**Supplementary Appendix A: Table 2**).

**Supplementary Appendix A: Table 1.** Variable importance produced by the generalized random forest.

| **Characteristics and category** | **Employment change** | **Earnings change** |
| --- | --- | --- |
| Age band | **18.2%** | **6.7%** |
| Country of birth: UK | 1.1% | 2.2% |
| Deprivation | 1.6% | 2.4% |
| Deprivation: missing | 1.4% | 0.0% |
| Diagnosis: Depression | 1.6% | 1.9% |
| Diagnosis: Generalized anxiety disorder | 1.4% | 1.7% |
| Diagnosis: Missing or not stated | 0.9% | 2.0% |
| Diagnosis: Mixed anxiety and depressive disorder | 0.5% | 1.9% |
| Diagnosis: Obsessive compulsive disorder | 0.1% | 0.0% |
| Diagnosis: Other anxiety or stress related disorder | 0.4% | 1.9% |
| Diagnosis: Panic disorder | 0.8% | 1.5% |
| Diagnosis: Post traumatic stress disorder | 0.5% | 1.3% |
| Diagnosis: Social phobias | 0.5% | 0.1% |
| Diagnosis: Specific (isolated) phobias | 0.1% | 0.0% |
| Disability | 0.9% | 1.4% |
| Ethnicity: Black | 0.9% | 2.4% |
| Ethnicity: Mixed | 1.2% | 0.1% |
| Ethnicity: Other | 0.6% | 0.0% |
| Ethnicity: White | 0.8% | 2.1% |
| Medication: Not prescribed | 0.7% | 2.2% |
| Medication: Prescribed, not taking | 0.7% | 1.6% |
| Medication: Prescribed, taking | 0.6% | 2.1% |
| First therapy quarter | 3.4% | **3.4%** |
| First therapy year | 1.5% | **3.0%** |
| Dependent children | 2.0% | **3.1%** |
| Dependent children: missing | 1.1% | 0.0% |
| Highest qualification: Level 1: 1 to 4 GCSEs grade A* to C, Any GCSEs at other grades, O levels or CSEs (any grades), 1 AS level, NVQ level 1, Foundation GNVQ, Basic or Essential Skills | 0.7% | 1.6% |
| Highest qualification: Level 2: 5 or more GCSEs (A* to C or 9 to 4), O levels (passes), CSEs (grade 1), School Certification, 1 A level, 2 to 3 AS levels, VCEs, Intermediate or Higher Diploma, Welsh Baccalaureate Intermediate Diploma, NVQ level 2, Intermediate GNVQ, City and Guilds Craft, BTEC First or General Diploma, RSA Diploma | 0.8% | 1.7% |
| Highest qualification: Level 3: 2 or more A levels or VCEs, 4 or more AS levels, Higher School Certificate, Progression or Advanced Diploma, Welsh Baccalaureate Advance Diploma, NVQ level 3; Advanced GNVQ, City and Guilds Advanced Craft, ONC, OND, BTEC National, RSA Advanced Diploma | 1.6% | 2.2% |
| Highest qualification: Level 4 and above: degree (BA, BSc), higher degree (MA, PhD, PGCE), NVQ level 4 to 5, HNC, HND, RSA Higher Diploma, BTEC Higher level, professional qualifications (for example, teaching, nursing, accountancy) | 1.9% | 2.5% |
| Highest qualification: No qualifications | 0.9% | 1.1% |
| Highest qualification: Other: vocational or work-related qualifications, other qualifications achieved in England or Wales, qualifications achieved outside England or Wales (equivalent not stated or unknown) | 0.7% | 1.9% |
| NS-SEC: Higher managerial administrative and professional occupations | 0.3% | **2.9%** |
| NS-SEC: Intermediate occupations | 0.5% | 1.5% |
| NS-SEC: Long term unemployed | 0.7% | 1.1% |
| NS-SEC: Lower managerial administrative and professional occupations | 0.6% | 2.5% |
| NS-SEC: Lower supervisory and technical occupations | 0.6% | 1.3% |
| NS-SEC: Never worked | 0.5% | 0.7% |
| NS-SEC: Routine occupations | 0.6% | 1.3% |
| NS-SEC: Semi routine occupations | 1.8% | 1.2% |
| NS-SEC: Small employers and own account workers | 0.6% | 1.4% |
| Employment status: Long term sick disabled | **4.4%** | 1.1% |
| Employment status: Missing or not stated | 1.0% | 1.4% |
| Employment status: Student | **10.2%** | 0.0% |
| Employment status: Not working, not seeking work | 0.9% | 1.0% |
| Employment status: Not working, seeking work | **14.3%** | 1.6% |
| Region: East of England | 0.5% | 1.9% |
| Region: London | 0.8% | 2.6% |
| Region: North East | 0.3% | 1.7% |
| Region: North West | 0.7% | 1.3% |
| Region: South East | 0.5% | 1.8% |
| Region: South West | **4.2%** | 1.8% |
| Region: West Midlands | 0.7% | 1.9% |
| Region: Yorkshire and The Humber | 0.6% | 2.1% |
| Sex: Males | 0.7% | 2.2% |
| Source of referral: Other | 0.6% | 1.4% |
| Source of referral: Primary Health Care | 0.7% | 2.1% |
| Source of referral: Self-referral | 0.9% | 2.4% |
| Therapy intensity: Low intensity | 1.6% | 1.9% |
| Therapy intensity: Missing | 0.3% | 0.0% |
|  |  |  |
| Notes: |  |  |
| 1. Five highest variables for each outcome are shown in bold. |  |  |

**Supplementary Appendix A: Table 2.** Effects of NHS TT treatment completion on monthly earnings (£) for those in paid employment at the time of first therapy

| **Time since first therapy  (in years)** | **Outcome measure** | **Estimate** | **Standard error** | **p value** | **Lower confidence limit** | **Upper confidence limit** |
| --- | --- | --- | --- | --- | --- | --- |
| -4 | Monthly earnings (£) | 0.4 | 4.6 | 0.937 | -8.6 | 9.3 |
| -3 | Monthly earnings (£) | -0.5 | 3.4 | 0.878 | -7.1 | 6.1 |
| -2 | Monthly earnings (£) | 2.0 | 2.5 | 0.426 | -3.0 | 7.0 |
| -1 | Monthly earnings (£) | -0.2 | 1.9 | 0.905 | -4.0 | 3.5 |
| 1 | Monthly earnings (£) | -3.7 | 2.2 | 0.092 | -8.0 | 0.6 |
| 2 | Monthly earnings (£) | -0.7 | 2.8 | 0.802 | -6.3 | 4.9 |
| 3 | Monthly earnings (£) | -6.5 | 3.2 | 0.046 | -12.8 | -0.1 |
| 4 | Monthly earnings (£) | -10.3 | 3.7 | 0.005 | -17.5 | -3.1 |
| 5 | Monthly earnings (£) | -13.6 | 4.4 | 0.002 | -22.3 | -5.0 |
| 6 | Monthly earnings (£) | -20.4 | 5.8 | 0 | -31.8 | -9.0 |
| 7 | Monthly earnings (£) | -27.8 | 10.8 | 0.01 | -49.0 | -6.6 |

The results from the data-driven GRF approach also support findings from the main analysis. Age band was highlighted as the most significant variable for both outcomes suggesting this variable is crucial in explaining the variations in employment outcomes. This is likely driven by the relationship between age and employment status during different stages of life^3^ as well as how well different groups respond to treatment. This might also reflect young people entering the workforce for the first time, salary and career progression driven by work experience, increased workplace stress over time^4^ or transition to retirement. The decreasing and negative effects on monthly earnings in the employed population might be explained by individuals reducing their working hours to facilitate improvements in mental health, avoid work related burn-out following a successful psychological therapy or moving to a job that makes them more happy but pays less^4,5^.

# **Supplementary Appendix B:** Sensitivity analysis:

Methods

We conducted several sensitivity analyses. For sensitivity analysis 1, we re-ran the models swapping our exposed group to those participants who had either reliably recovered or improved. The non-exposed group comprised participants who had no change or deterioration of their mental health symptoms following NHSTT treatment. For this analysis participants with missing recovery types were removed (N=5,000). For sensitivity analysis 2, we compared the completed treatment group to individuals who were referred to NHS Talking Therapies but left before assessment and treatment.

We also conducted analysis on monthly records for a random sample of participants (due to processing restrictions) to account for potential seasonal patterns masked by aggregated quarterly data (Sensitivity analysis 3). Additionally, to examine the extent to which age (at the time of referral) affected the labour market effects, we tested models with different inclusion criteria for age (18 years old at the time of referral with prior follow-up back to 16 years) (Sensitivity analysis 4).

Results: Sensitivity analysis 1

The first part of the sensitivity analyses assessed the impact of swapping the exposure groups. The exposed group for this analysis includes individuals who had reliable recovery or reliable improvement (N=645,381) and the non-exposed group includes individuals who had no change or reliable deterioration in their mental health symptoms (N=191,746) (See **Supplementary Appendix B: Table 1** for summary statistics. and **Supplementary Appendix B: Table 2** for average follow up time in quarters). The exposed group earned more on average than the non-exposed group before and after treatment, while the IPWs made the earnings trajectories more parallel (**Supplementary Appendix B: Figure 1).**

**Supplementary Appendix B: Figure 1.** Average monthly pay and average monthly pay with IPWs broken down by recovery exposure groups


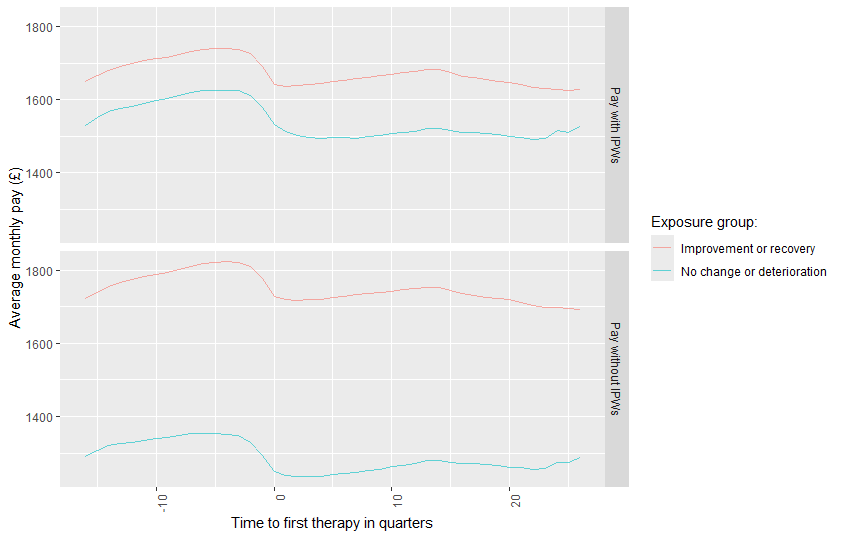


**Supplementary Appendix B: Table 1.** Sociodemographic summary statistics between recovery exposure groups (sensitivity analyses).

| **Characteristic** | **Category** | **Improvement or recovery** | **No change or deterioration** |
| --- | --- | --- | --- |
| Age band | 25-34 | 219902 (34.1%) | 69145 (36.1%) |
|  | 35-44 | 184008 (28.5%) | 53260 (27.8%) |
|  | 45-54 | 167185 (25.9%) | 48228 (25.2%) |
|  | 55-60 | 74286 (11.5%) | 21113 (11.0%) |
| Country of birth | Non-UK | 57812 (9.0%) | 18475 (9.6%) |
|  | UK | 587569 (91.0%) | 173271 (90.4%) |
| Dependent children | Missing or not stated | 7699 (1.2%) | 2355 (1.2%) |
|  | No children | 331681 (51.4%) | 94925 (49.5%) |
|  | One child | 145764 (22.6%) | 44370 (23.1%) |
|  | Three or more children | 45464 (7.0%) | 17133 (8.9%) |
|  | Two children | 114773 (17.8%) | 32963 (17.2%) |
| Deprivation | Deprived, 1 dimension | 184536 (28.6%) | 58373 (30.4%) |
|  | Deprived, 2 dimensions | 82045 (12.7%) | 35603 (18.6%) |
|  | Deprived, 3 dimensions | 26857 (4.2%) | 15347 (8.0%) |
|  | Deprived, 4 dimensions | 2597 (0.4%) | 1672 (0.9%) |
|  | Missing or not stated | 7699 (1.2%) | 2355 (1.2%) |
|  | Not deprived | 341647 (52.9%) | 78396 (40.9%) |
| Diagnosis | Agoraphobia | 3524 (0.5%) | 1776 (0.9%) |
|  | Depression | 243868 (37.8%) | 76744 (40%) |
|  | Generalized anxiety disorder | 163218 (25.3%) | 39153 (20.4%) |
|  | Missing or not stated | 71222 (11.0%) | 24074 (12.6%) |
|  | Mixed anxiety and depressive disorder | 64169 (9.9%) | 20421 (10.7%) |
|  | Obsessive-compulsive disorder | 13133 (2.0%) | 4111 (2.1%) |
|  | Other anxiety or stress related disorder | 28062 (4.3%) | 7101 (3.7%) |
|  | Panic disorder [episodic paroxysmal anxiety] | 16735 (2.6%) | 4782 (2.5%) |
|  | Post-traumatic stress disorder | 23599 (3.7%) | 8352 (4.4%) |
|  | Social phobias | 12986 (2.0%) | 4205 (2.2%) |
|  | Specific (isolated) phobias | 4865 (0.8%) | 1027 (0.5%) |
| Disability | Activities limited a little | 52662 (8.2%) | 21923 (11.4%) |
|  | Activities limited a lot | 31290 (4.8%) | 18111 (9.4%) |
|  | Not disabled or activities not limited | 561429 (87.0%) | 151712 (79.1%) |
| Employment status | Employed | 445826 (69.1%) | 104270 (54.4%) |
|  | Long-term sick/disabled | 39234 (6.1%) | 23501 (12.3%) |
|  | Missing or not stated | 59794 (9.3%) | 21560 (11.2%) |
|  | Student | 6884 (1.1%) | 2402 (1.3%) |
|  | Unemployed, not seeking work | 47100 (7.3%) | 17639 (9.2%) |
|  | Unemployed, seeking work | 46543 (7.2%) | 22374 (11.7%) |
| Ethnicity | Asian | 29200 (4.5%) | 10008 (5.2%) |
|  | Black | 15761 (2.4%) | 4981 (2.6%) |
|  | Mixed | 13029 (2.0%) | 4511 (2.4%) |
|  | Other | 3969 (0.6%) | 1626 (0.8%) |
|  | White | 583422 (90.4%) | 170620 (89.0%) |
| Highest qualification | Apprenticeship | 11586 (1.8%) | 3286 (1.7%) |
|  | Level 1 | 98577 (15.3%) | 34964 (18.2%) |
|  | Level 2 | 129015 (20.0%) | 39724 (20.7%) |
|  | Level 3 | 127998 (19.8%) | 34235 (17.9%) |
|  | Level 4 and above | 213201 (33.0%) | 47561 (24.8%) |
|  | No qualifications | 48181 (7.5%) | 25897 (13.5%) |
|  | Other | 16823 (2.6%) | 6079 (3.2%) |
| Medication (Psychotropic Medication) | Missing or not stated | 30300 (4.7%) | 10707 (5.6%) |
|  | Not prescribed | 274227 (42.5%) | 69354 (36.2%) |
|  | Prescribed, not taking | 28878 (4.5%) | 9303 (4.9%) |
|  | Prescribed, taking | 311976 (48.3%) | 102382 (53.4%) |
| NS-SEC | Full-Time students | 63245 (9.8%) | 18149 (9.5%) |
|  | Higher managerial, administrative and professional occupations | 55471 (8.6%) | 11878 (6.2%) |
|  | Intermediate occupations | 112223 (17.4%) | 29790 (15.5%) |
|  | Long-term unemployed | 14146 (2.2%) | 6859 (3.6%) |
|  | Lower managerial, administrative and professional occupations | 155878 (24.2%) | 36204 (18.9%) |
|  | Lower supervisory and technical occupations | 36468 (5.7%) | 11158 (5.8%) |
|  | Never worked | 18507 (2.9%) | 11032 (5.8%) |
|  | Routine occupations | 53751 (8.3%) | 20760 (10.8%) |
|  | Semi-routine occupations | 101437 (15.7%) | 35591 (18.6%) |
|  | Small employers and own account workers | 34255 (5.3%) | 10325 (5.4%) |
| Number of treatment sessions | 2 | 47891 (7.4%) | 49367 (25.7%) |
|  | 3 to 5 | 198051 (30.7%) | 70337 (36.7%) |
|  | 6 to 8 | 207884 (32.2%) | 37051 (19.3%) |
|  | 9 to 15 | 142908 (22.1%) | 25589 (13.3%) |
|  | 16 or more | 48647 (7.5%) | 9402 (4.9%) |
| Region | East Midlands | 66734 (10.3%) | 18596 (9.7%) |
|  | East of England | 63984 (9.9%) | 19722 (10.3%) |
|  | London | 88255 (13.7%) | 26678 (13.9%) |
|  | North East | 44039 (6.8%) | 13652 (7.1%) |
|  | North West | 88231 (13.7%) | 28816 (15.0%) |
|  | South East | 110030 (17.0%) | 28764 (15.0%) |
|  | South West | 58420 (9.1%) | 19499 (10.2%) |
|  | West Midlands | 59319 (9.2%) | 16386 (8.5%) |
|  | Yorkshire and The Humber | 66369 (10.3%) | 19633 (10.2%) |
| Sex | Females | 430580 (66.7%) | 129872 (67.7%) |
|  | Males | 214801 (33.3%) | 61874 (32.3%) |
| Source of referral | Missing or not stated | 1839 (0.3%) | 665 (0.3%) |
|  | Other | 36072 (5.6%) | 14694 (7.7%) |
|  | Primary Health Care | 125052 (19.4%) | 41768 (21.8%) |
|  | Self-referral | 482418 (74.7%) | 134619 (70.2%) |
| Therapy intensity | High intensity | 417881 (64.7%) | 119088 (62.1%) |
|  | Low intensity | 225634 (35.0%) | 71952 (37.5%) |
|  | Missing | 1866 (0.3%) | 706 (0.4%) |

**Supplementary Appendix B: Table 2.** Follow-up times (in quarters) for the recovery types exposed and non-exposed groups

| **Exposure group** | **Minimum follow-up pre-treatment (in calendar quarters)** | **Maximum follow-up pre-treatment (in calendar quarters)** | **Average follow-up pre-treatment (in calendar quarters)** | **Minimum follow-up post-treatment (in calendar quarters)** | **Maximum follow-up post-treatment (in calendar quarters)** | **Average follow-up post-treatment (in calendar quarters)** |
| --- | --- | --- | --- | --- | --- | --- |
| Improvement or recovery (2nd exposed group) | -8 | -16 | -14.2 | 1 | 26 | 19.7 |
| No change or deterioration (2nd non-exposed group) | -8 | -16 | -14.1 | 1 | 26 | 19.8 |

**Supplementary Appendix B: Figure 2.** Effects of positive recovery types after psychological therapies treatment on (A) monthly earnings and (B) probability of being a paid employee. The non-exposed group includes participants with no change or deterioration in their mental health symptoms.


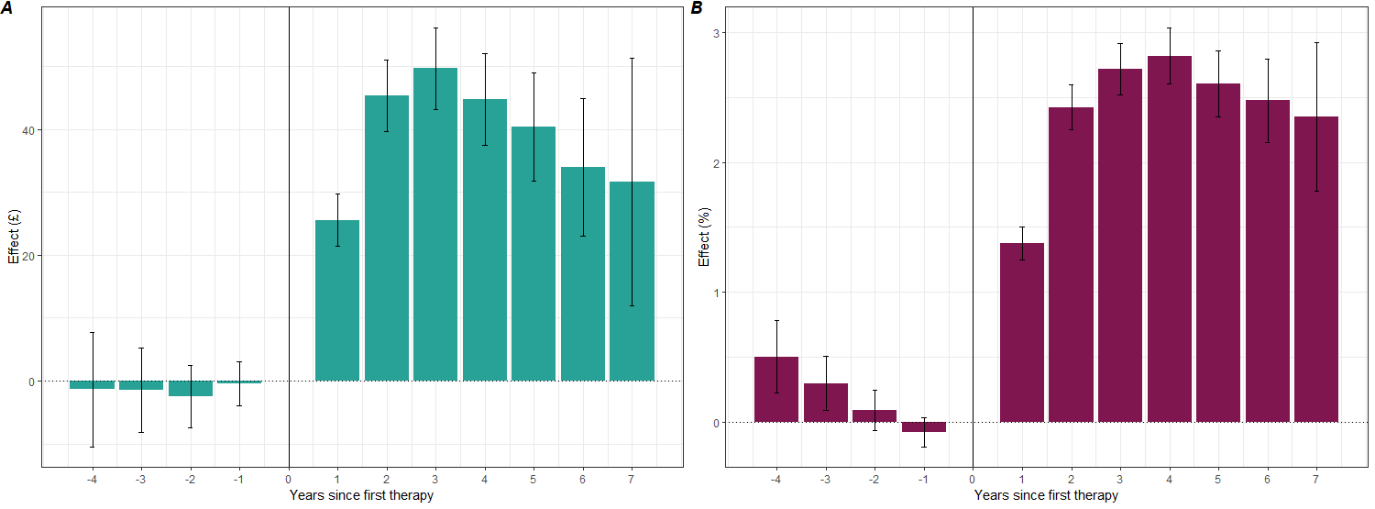


Reliable recovery and reliable improvement of mental health symptoms were strongly associated with improved monthly earnings and employee status. The largest effect on monthly earnings was found in year three after treatment and was equal to £49.7 [95% CI 43.2-56.2] more per month; the statistically significant increase relative to pre-treatment was sustained at £31.7 [95% CI 11.9-51.4] in year seven (**Supplementary Appendix B: Figure 2**). Similarly, the probability of being a paid employee increased by 2.8 p.p. [95% CI 2.6-3.0] four years after therapy (largest effect). From year two onwards, the probability of being a paid employee was sustained and by year seven the increase reached 2.3 p.p. [95% CI 1.8-2.9] (**Supplementary Appendix B: Figure 2**). For breakdowns by age and number of treatment sessions, see **Supplementary Appendix B: Table 3**.

**Supplementary Appendix B: Table 3.** Effects of mental health recovery or improvement after psychological therapies treatment on probability of being a paid employee by (A) age and (B) number of treatment sessions.

The non-exposed group includes participants with no change or deterioration.

| **Characteristic** | **Breakdown** | **Time since first therapy (in years)** | | | | | | | | | | |
| --- | --- | --- | --- | --- | --- | --- | --- | --- | --- | --- | --- | --- |
|  |  | **-4** | **-3** | **-2** | **-1** | **1** | **2** | **3** | **4** | **5** | **6** | **7** |
| **Age band** | **25-34** | 0.7**  (0.2-1.2) | 0.6**  (0.2-0.9) | 0.2  (-0.1-0.5) | 0.0  (-0.2-0.3) | 1.1**  (0.9-1.4) | 2.1**  (1.8-2.4) | 2.5**  (2.2-2.9) | 2.7**  (2.4-3.1) | 2.4**  (1.9-2.8) | 2.3**  (1.7-2.8) | 2.0**  (1.0-3.0) |
|  | **35-44** | 0.1  (-0.4-0.7) | 0.0  (-0.3-0.4) | -0.1  (-0.4-0.2) | -0.2  (-0.4-0) | 1.6**  (1.4-1.8) | 2.7**  (2.3-3) | 2.9**  (2.6-3.3) | 3.1**  (2.7-3.5) | 3.2**  (2.8-3.7) | 3.1**  (2.5-3.7) | 2.7**  (1.7-3.7) |
|  | **45-54** | 0.5  (0.0-1.0) | 0.1  (-0.2-0.5) | 0.1  (-0.2-0.4) | -0.2  (-0.4-0) | 1.6**  (1.3-1.8) | 2.8**  (2.4-3.1) | 3.0**  (2.6-3.4) | 3.1**  (2.6-3.5) | 2.9**  (2.4-3.4) | 2.9**  (2.2-3.5) | 2.9**  (1.8-4.0) |
|  | **55-60** | 0.1  (-0.7-0.9) | -0.1  (-0.7-0.5) | -0.1  (-0.5-0.4) | -0.2  (-0.5-0.1) | 1.1**  (0.7-1.5) | 2.1**  (1.6-2.7) | 2.0**  (1.4-2.7) | 1.7**  (1.0-2.5) | 1.0*  (0.2-1.9) | 0.4  (-0.8-1.5) | 1.0  (-1.2-3.2) |
| **Number of treatment sessions** | **2** | -0.3  (-1.0-0.4) | -0.3  (-0.8-0.2) | -0.3  (-0.7-0.1) | -0.2  (-0.5-0.1) | 1.2**  (0.9-1.5) | 1.7**  (1.3-2.1) | 1.9**  (1.4-2.4) | 2.1**  (1.6-2.7) | 1.4**  (0.8-2.0) | 1.4**  (0.6-2.2) | 1.4  (-0.1-2.8) |
|  | **3 to 5** | 0.3  (-0.2-0.7) | 0.2  (-0.2-0.5) | 0.1  (-0.2-0.3) | -0.2  (-0.4-0.0) | 1.5**  (1.3-1.8) | 2.5**  (2.2-2.8) | 2.7**  (2.3-3.0) | 2.7**  (2.4-3.1) | 2.5**  (2.0-2.9) | 2.3**  (1.7-2.8) | 2.2**  (1.2-3.2) |
|  | **6 to 8** | 0.8**  (0.2-1.4) | 0.4  (-0.1-0.8) | 0.1  (-0.3-0.4) | -0.1  (-0.3-0.1) | 1.7**  (1.4-2.0) | 2.7**  (2.3-3.1) | 3.0**  (2.6-3.5) | 3.0**  (2.6-3.5) | 3.0**  (2.4-3.5) | 3.0**  (2.3-3.7) | 2.7**  (1.5-4.0) |
|  | **9 to 15** | 0.8*  (0.1-1.6) | 0.3  (-0.3-0.9) | 0.0  (-0.4-0.4) | -0.1  (-0.4-0.2) | 1.6** (1.3-1.9) | 3.0**  (2.6-3.5) | 3.1**  (2.6-3.7) | 3.3**  (2.7-3.9) | 3.2**  (2.6-3.9) | 2.9**  (2.0-3.7) | 2.9**  (1.4-4.5) |
|  | **16 or more** | 0.9  (-0.4-2.2) | 0.3  (-0.6-1.2) | -0.2  (-0.9-0.5) | -0.5  (-1.0-0.0) | 1.6**  (1.0-2.1) | 3.3**  (2.5-4.0) | 3.5**  (2.6-4.3) | 3.5**  (2.5-4.4) | 3.4**  (2.3-4.5) | 3.6**  (2.2-5.0) | 1.8  (-0.7-4.3) |

Notes:

1. * = p value <0.05 and ** = p value <0.01
2. The dotted line marks the intervention point; data to the left of the line correspond to the pre-intervention period, while data to the right reflect the post-intervention period.

Results: Sensitivity analysis 2

The exposed group for this analysis includes individuals who completed treatment (N=296,650), and the non-exposed group includes individuals who were referred to NHS Talking Therapies but did not receive assessment or treatment (N=237,727). Sample of 50% was taken from both groups to reduce the size of the dataset to improve memory related processing issues.

The exposed group earned more on average than the non-exposed group before and after treatment (**Supplementary Appendix B: Figure 3**). The IPWs improved the trajectories of earnings, but did not fully account for different trends between both groups. As the non-exposed group did not enter treatment, information on diagnosis, self-reported employment status, therapy intensity, psychotropic medication use, and source of referral were not included in the IPW model. Only Census variables were included.

**Supplementary Appendix B: Figure 3**. Average monthly pay and average monthly pay with IPWs broken down by completed and referred exposure groups


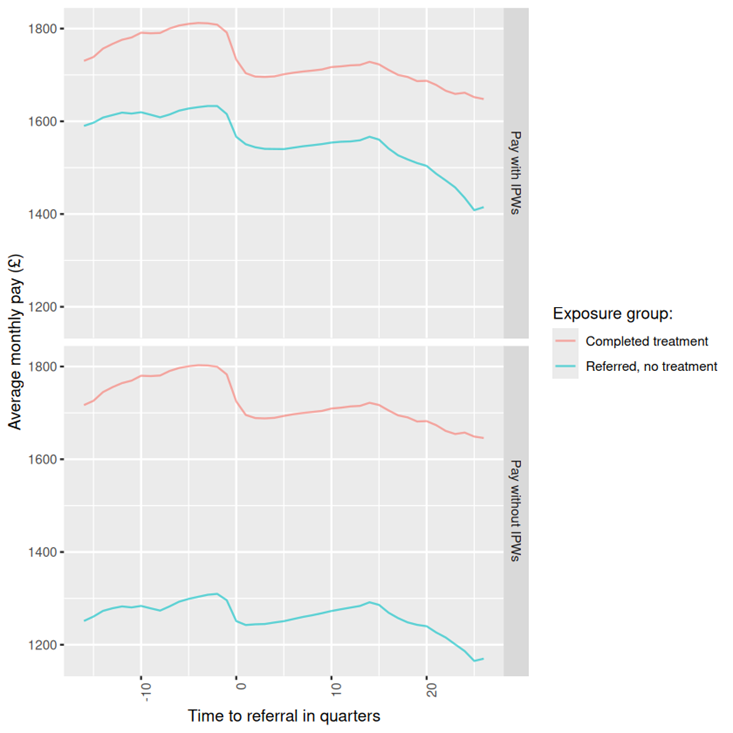


**Supplementary Appendix B: Figure 4.** Effects of completing treatment compared to not receiving treatment on (A) monthly earnings and (B) probability of being a paid employee. The non-exposed group includes participants who were referred to NHSTT but left before assessment and treatment


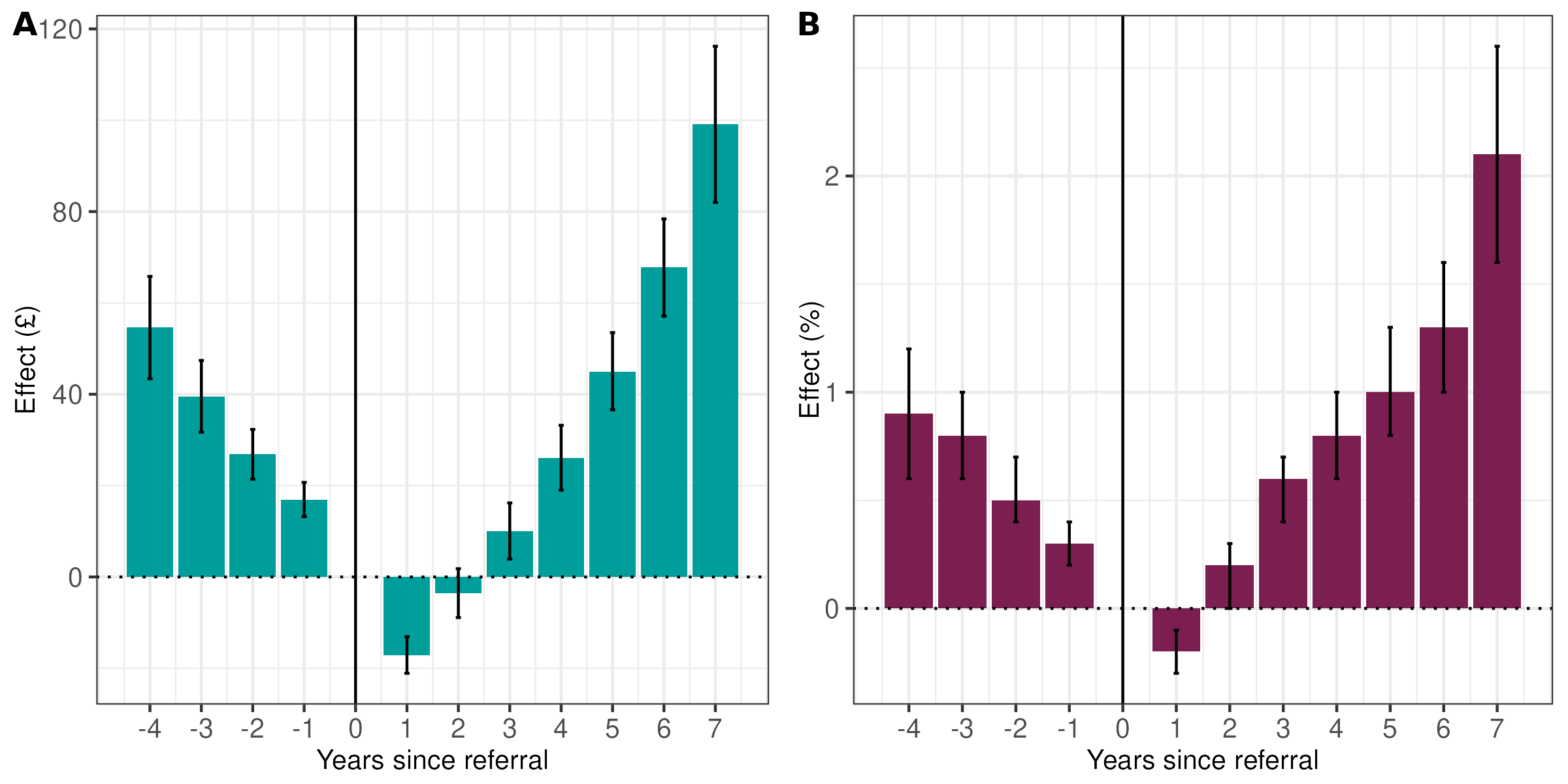


The largest statistically significant effect on monthly earnings was found in year seven and was equal to £99.1 [95% CI 82.0-116.2] more per month (**Supplementary Appendix B: Figure 4**). Similarly, the probability of being a paid employee increased by 2.1 p.p. [95% CI 1.6-2.6] seven years after therapy (largest statistically significant effect) (Supplementary Appendix B: Figure 4). It is important to point out the diverging trends before treatment between the two exposure groups. The groups seem to be statistically very different from each other which leads to the violation of the parallel trend assumption. Due to diverging trends in outcome before treatment, we cannot accurately attribute the changes in employment and earnings to effects of NHSTT as they could be driven by the changes in the differentiating outcome trends.

Results: Sensitivity analysis 3 and 4

The results from analysis on monthly records for a random sample of participants were in line with those from aggregated quarterly data (**Supplementary Appendix B: Table 5**). The effect size of the overall models was larger when we changed the inclusion criteria for age (18 years at time of referral with prior follow-up back to 16 years), likely driven by younger participants entering the workforce for the first time (**Supplementary Appendix B: Table 5**).

**Supplementary Appendix B: Table 5.** Other sensitivity analysis results.

| **Sensitivity analysis type** | **Time since first therapy (in years)** | **Outcome measure** | **Estimate** | **Standard error** | **p value** | **Lower confidence limit** | **Upper confidence limit** |
| --- | --- | --- | --- | --- | --- | --- | --- |
| Change of age inclusion criteria, 18-years-old at the time of referral with prior follow-up back to 16 | -4 | Monthly earnings (£) | -13.8 | 4.0 | < 0.01 | -21.6 | -5.9 |
|  | -3 | Monthly earnings (£) | -9.2 | 2.8 | < 0.01 | -14.8 | -3.7 |
|  | -2 | Monthly earnings (£) | -2.7 | 2.0 | 0.19 | -6.6 | 1.3 |
|  | -1 | Monthly earnings (£) | -0.9 | 1.4 | 0.50 | -3.7 | 1.8 |
|  | 1 | Monthly earnings (£) | 6.8 | 1.6 | < 0.01 | 3.7 | 9.9 |
|  | 2 | Monthly earnings (£) | 21.7 | 2.2 | < 0.01 | 17.4 | 26.0 |
|  | 3 | Monthly earnings (£) | 28.4 | 2.5 | < 0.01 | 23.4 | 33.3 |
|  | 4 | Monthly earnings (£) | 32.5 | 2.9 | < 0.01 | 26.9 | 38.1 |
|  | 5 | Monthly earnings (£) | 33.4 | 3.4 | < 0.01 | 26.8 | 40.0 |
|  | 6 | Monthly earnings (£) | 33.6 | 4.3 | < 0.01 | 25.2 | 42.0 |
|  | 7 | Monthly earnings (£) | 37.5 | 6.8 | < 0.01 | 24.2 | 50.8 |
|  | -4 | Probability of being a paid employee (%) | 0.2 | 0.1 | 0.29 | -0.1 | 0.4 |
|  | -3 | Probability of being a paid employee (%) | 0.2 | 0.1 | 0.13 | 0.0 | 0.4 |
|  | -2 | Probability of being a paid employee (%) | 0.2 | 0.1 | < 0.05 | 0.0 | 0.3 |
|  | -1 | Probability of being a paid employee (%) | 0.1 | 0.1 | 0.18 | 0.0 | 0.2 |
|  | 1 | Probability of being a paid employee (%) | 0.4 | 0.1 | < 0.01 | 0.3 | 0.6 |
|  | 2 | Probability of being a paid employee (%) | 1.2 | 0.1 | < 0.01 | 1.1 | 1.4 |
|  | 3 | Probability of being a paid employee (%) | 1.6 | 0.1 | < 0.01 | 1.5 | 1.8 |
|  | 4 | Probability of being a paid employee (%) | 1.8 | 0.1 | < 0.01 | 1.7 | 2.0 |
|  | 5 | Probability of being a paid employee (%) | 1.8 | 0.1 | < 0.01 | 1.6 | 2.0 |
|  | 6 | Probability of being a paid employee (%) | 1.7 | 0.1 | < 0.01 | 1.5 | 2.0 |
|  | 7 | Probability of being a paid employee (%) | 2.0 | 0.2 | < 0.01 | 1.5 | 2.4 |
| Monthly records on 20% sample instead of quarterly records | -4 | Monthly earnings (£) | 4.9 | 9.7 | 0.61 | -14.1 | 23.9 |
|  | -3 | Monthly earnings (£) | 7.0 | 7.1 | 0.33 | -7.0 | 20.9 |
|  | -2 | Monthly earnings (£) | 9.2 | 5.2 | 0.08 | -0.9 | 19.4 |
|  | -1 | Monthly earnings (£) | 4.3 | 3.7 | 0.25 | -3.0 | 11.5 |
|  | 1 | Monthly earnings (£) | 3.2 | 4.3 | 0.47 | -5.4 | 11.7 |
|  | 2 | Monthly earnings (£) | 15.3 | 5.9 | < 0.05 | 3.6 | 26.9 |
|  | 3 | Monthly earnings (£) | 16.5 | 6.8 | < 0.05 | 3.3 | 29.8 |
|  | 4 | Monthly earnings (£) | 17.0 | 7.6 | < 0.05 | 2.1 | 31.9 |
|  | 5 | Monthly earnings (£) | 20.5 | 8.9 | < 0.05 | 3.0 | 38.0 |
|  | 6 | Monthly earnings (£) | 11.4 | 11.4 | 0.32 | -10.9 | 33.7 |
|  | 7 | Monthly earnings (£) | 20.4 | 19.0 | 0.28 | -16.8 | 57.6 |
|  | -4 | Probability of being a paid employee (%) | 0.2 | 0.0 | 0.46 | -0.4 | 0.8 |
|  | -3 | Probability of being a paid employee (%) | 0.3 | 0.0 | 0.22 | -0.2 | 0.7 |
|  | -2 | Probability of being a paid employee (%) | 0.4 | 0.0 | < 0.05 | 0.0 | 0.7 |
|  | -1 | Probability of being a paid employee (%) | 0.1 | 0.0 | 0.32 | -0.1 | 0.4 |
|  | 1 | Probability of being a paid employee (%) | 0.5 | 0.0 | < 0.05 | 0.2 | 0.7 |
|  | 2 | Probability of being a paid employee (%) | 1.2 | 0.0 | < 0.01 | 0.8 | 1.6 |
|  | 3 | Probability of being a paid employee (%) | 1.4 | 0.0 | < 0.01 | 1.0 | 1.8 |
|  | 4 | Probability of being a paid employee (%) | 1.4 | 0.0 | < 0.01 | 1.0 | 1.9 |
|  | 5 | Probability of being a paid employee (%) | 1.6 | 0.0 | < 0.01 | 1.0 | 2.1 |
|  | 6 | Probability of being a paid employee (%) | 1.3 | 0.0 | < 0.01 | 0.7 | 2.0 |
|  | 7 | Probability of being a paid employee (%) | 1.3 | 0.0 | < 0.05 | 0.3 | 2.4 |

**References**

1. Athey S, Tibshirani J, Wager S. Generalised random forests. Ann Stat. 2019;47(2):1148–78.

2. Nie X, Wager S. Quasi-oracle estimation of heterogenous treatment effects. Briometrika. 2021;108(2):299=319.

3. Härkönen J, Bihagen E. Occupational attainment and career progression in Sweden. Eur Soc. 2011;13(3):451–79.

4. Voglino G, Savatteri A, Gualano MR, Catozzi D, Rousset S, Boietti E, et al. How the reduction of working hours could influence health outcomes: a systematic review of published studies. BMJ Open [Internet]. 2022;12(4). Available from: https://bmjopen.bmj.com/content/12/4/e051131

5. Virtanen M, Ferrie JE, Singh-Manoux A, Shipley MJ, Stansfeld SA, Marmot MG, et al. Long working hours and symptoms of anxiety and depression: a 5-year follow-up of the Whitehall II study. Psychol Med [Internet]. 2011/02/18. 2011;41(12):2485–94. Available from: https://www.cambridge.org/core/product/B19F5D1AC436F10E5954C8ADBBE2AC57
